# Copy Number Variant Risk Scores Associated With Cognition, Psychopathology, and Brain Structure in Youths in the Philadelphia Neurodevelopmental Cohort

**DOI:** 10.1001/jamapsychiatry.2022.1017

**Published:** 2022-05-11

**Authors:** Aaron Alexander-Bloch, Guillaume Huguet, Laura M. Schultz, Nicholas Huffnagle, Sebastien Jacquemont, Jakob Seidlitz, Zohra Saci, Tyler M. Moore, Richard A. I. Bethlehem, Josephine Mollon, Emma K. Knowles, Armin Raznahan, Alison Merikangas, Barbara H. Chaiyachati, Harshini Raman, J. Eric Schmitt, Ran Barzilay, Monica E. Calkins, Russel T. Shinohara, Theodore D. Satterthwaite, Ruben C. Gur, David C. Glahn, Laura Almasy, Raquel E. Gur, Hakon Hakonarson, Joseph Glessner

**Affiliations:** 1 Department of Child and Adolescent Psychiatry and Behavioral Science, Children’s Hospital of Philadelphia, Philadelphia, Pennsylvania; 2 The Lifespan Brain Institute, Children’s Hospital of Philadelphia and Penn Medicine, University of Pennsylvania, Philadelphia; 3 Neurodevelopment and Psychosis Section, Department of Psychiatry, University of Pennsylvania, Philadelphia; 4 Department of Pediatrics, University of Montreal, Montreal, Quebec, Canada; 5 Research Center of the Sainte-Justine University Hospital, Montreal, Quebec, Canada; 6 Department of Biomedical and Health Informatics, Children’s Hospital of Philadelphia, Philadelphia, Pennsylvania; 7 Department of Psychiatry, University of Cambridge, Cambridge, United Kingdom; 8 Department of Psychiatry, Boston Children’s Hospital, Harvard Medical School, Boston, Massachusetts; 9 Section on Developmental Neurogenomics, National Institute of Mental Health, Bethesda, Maryland; 10 Department of Genetics, University of Pennsylvania, Philadelphia; 11 Center for Applied Genomics, Children’s Hospital of Philadelphia, Philadelphia, Pennsylvania; 12 Department of Pediatrics, University of Pennsylvania, Philadelphia; 13 Wellesley College, Wellesley, Massachusetts; 14 Department of Radiology, University of Pennsylvania, Philadelphia, Pennsylvania; 15 Penn Statistics in Imaging and Visualization Center, University of Pennsylvania, Philadelphia; 16 Department of Biostatistics, Epidemiology, and Informatics, University of Pennsylvania, Philadelphia; 17 Penn Center for Biomedical Image Computing and Analytics, University of Pennsylvania, Philadelphia; 18 Penn Lifespan Informatics and Neuroimaging Center, University of Pennsylvania, Philadelphia

## Abstract

**Importance:**

Psychiatric and cognitive phenotypes have been associated with a range of specific, rare copy number variants (CNVs). Moreover, IQ is strongly associated with CNV risk scores that model the predicted risk of CNVs across the genome. But the utility of CNV risk scores for psychiatric phenotypes has been sparsely examined.

**Objective:**

To determine how CNV risk scores, common genetic variation indexed by polygenic scores (PGSs), and environmental factors combine to associate with cognition and psychopathology in a community sample.

**Design, Setting, and Participants:**

The Philadelphia Neurodevelopmental Cohort is a community-based study examining genetics, psychopathology, neurocognition, and neuroimaging. Participants were recruited through the Children’s Hospital of Philadelphia pediatric network. Participants with stable health and fluency in English underwent genotypic and phenotypic characterization from November 5, 2009, through December 30, 2011. Data were analyzed from January 1 through July 30, 2021.

**Exposures:**

The study examined (1) CNV risk scores derived from models of burden, predicted intolerance, and gene dosage sensitivity; (2) PGSs from genomewide association studies related to developmental outcomes; and (3) environmental factors, including trauma exposure and neighborhood socioeconomic status.

**Main Outcomes and Measures:**

The study examined (1) neurocognition, with the Penn Computerized Neurocognitive Battery; (2) psychopathology, with structured interviews based on the Schedule for Affective Disorders and Schizophrenia for School-Age Children; and (3) brain volume, with magnetic resonance imaging.

**Results:**

Participants included 9498 youths aged 8 to 21 years; 4906 (51.7%) were female, and the mean (SD) age was 14.2 (3.7) years. After quality control, 18 185 total CNVs greater than 50 kilobases (10 517 deletions and 7668 duplications) were identified in 7101 unrelated participants genotyped on Illumina arrays. In these participants, elevated CNV risk scores were associated with lower overall accuracy on cognitive tests (standardized β = 0.12; 95% CI, 0.10-0.14; *P* = 7.41 × 10^−26^); lower accuracy across a range of cognitive subdomains; increased overall psychopathology; increased psychosis-spectrum symptoms; and higher deviation from a normative developmental model of brain volume. Statistical models of developmental outcomes were significantly improved when CNV risk scores were combined with PGSs and environmental factors.

**Conclusions and Relevance:**

In this study, elevated CNV risk scores were associated with lower cognitive ability, higher psychopathology including psychosis-spectrum symptoms, and greater deviations from normative magnetic resonance imaging models of brain development. Together, these results represent a step toward synthesizing rare genetic, common genetic, and environmental factors to understand clinically relevant outcomes in youth.

## Key Points

**Question:**

How do copy number variants (CNVs) combine with common genetic variants and environmental factors to help explain variability in cognition and psychopathology in a community sample?

**Findings:**

In this community-based cohort study including 9498 youths in the Philadelphia Neurodevelopmental Cohort, elevated CNV risk scores were associated with lower cognitive ability and more subtly associated with both higher overall psychopathology and higher psychosis-spectrum symptoms. Statistical models of cognitive and psychopathological outcomes were significantly improved when CNV risk scores were combined with polygenic scores and quantitative measures of environmental stress.

**Meaning:**

It is important to integrate rare genetic, common genetic, and environmental factors in investigations of clinically relevant developmental outcomes.

## Teaser

This community-based cohort study evaluates how copy number variant (CNV) risk scores, common genetic variation indexed by polygenic scores, and environmental factors combine to associate with cognition and psychopathology in the Philadelphia Neurodevelopmental Cohort (PNC).

## Introduction

Deletions or duplications of genomic segments known as copy number variants (CNVs) are major contributors to liability for complex diseases, including mental illness. Many so-called genomic disorders, historically characterized by sets of clinical features and now linked to specific recurrent CNVs, are associated with autism, schizophrenia, and intellectual disability.^1,2,3,4,5^ For example, up to 25% of individuals with 22q11.2 deletion syndrome develop schizophrenia.^6^ Multiple recurrent CNVs have also been associated with cognitive outcomes^7,8^ and depressive symptoms in adults.^9^ However, most clinically relevant CNVs are ultra-rare, with frequencies too low for sufficiently powered tests of genomewide association.^10^ In the clinical setting, screening children with neurodevelopmental disorders using chromosomal microarrays identifies potentially causal CNVs in 10% to 15% of cases.^11,12,13^ Yet the association of CNVs (especially nonrecurrent CNVs) with psychiatric morbidity has only been sparsely explored.

Despite continued advances, serious obstacles limit the diagnostic and prognostic potential of CNVs in psychiatry. Many CNVs exhibit variable penetrance and expressivity.^1^ This interindividual phenotypic variability highlights the importance of simultaneously considering other risk factors, including common genetic variation, environmental factors, and cumulative burden of multiple CNVs.^14,15,16^ Another limitation is that pathogenicity of ultra-rare CNVs is, in essence, binary in the clinical context (ie, disease causing or not), in contrast with continuous measures of symptomatology in contemporary psychiatric phenotyping.^17^ Notably, recent work leveraged annotations of haploinsufficient genes^18,19^ (genes whose function is sensitive to copy number loss) to derive CNV risk scores that predict IQ loss and autism risk for both recurrent and nonrecurrent CNVs.^20,21,22^ For example, IQ quantitative models estimated a negative effect size of 2.6 IQ points for deletions and 0.8 IQ points for duplications per unit of predicted haploinsufficiency intolerance, successfully predicting IQ in recurrent pathogenic CNVs.^21^ These studies motivate further research to characterize associations between CNV risk scores and dimensional measures of psychopathology as well as finer-grained measures of cognitive performance beyond IQ.

To advance work on quantitative models of CNV-related developmental outcomes, there are also compelling reasons to investigate a combined framework that integrates common genetic and environmental factors. It is well established that exposures to long-term and acute environmental stressors are strongly associated with interindividual variability in domains of cognition and psychopathology.^23,24,25,26^ Moreover, the multiple hit model of cumulative genetic and environmental impacts has support from numerous sources.^27,28,29,30^ Complementarily, the cumulative impact of common variants, as quantified by polygenic scores (PGSs), explains significant variance in many complex traits, eg, approximately 3% of variance in general intelligence (g)^31,32^ and up to 7% of liability for schizophrenia.^16^ Moreover, PGSs may have greater predictive power in at-risk individuals, including individuals with genomic disorders.^16^

In the present study, associations between CNVs and developmental outcomes were investigated in the Philadelphia Neurodevelopmental Cohort (PNC), a well-characterized community sample where CNVs have not previously been examined. The PNC included comprehensive clinical assessments, cognitive batteries, and brain magnetic resonance images (MRIs) during the critical period of adolescence.^33,34^ This allowed the present study to assess, in concert, subdomains of cognition and clinical symptomatology; neuroimaging phenotypes; common genetic variation in the form of PGSs; and environmental risk factors, eg, socioeconomic burden and history of trauma exposure. We aimed to (1) evaluate, in a large developmental cohort, the previously reported quantitative association between CNV risk scores and cognition; (2) examine associations between CNV risk scores and subdomains of clinical symptomatology as well as measures of deviation from typical brain development indexed by MRI; and (3) investigate models that integrate CNV risk scores with PGSs and environmental factors. We hypothesized that risk scores derived from integrating all CNV-associated genes, weighted by intolerance or dosage sensitivity scores,^35^ would be preferentially associated with cognitive and clinical symptom domains, combining with PGS and environmental factors to explain interindividual variation in a range of developmental outcomes.

## Methods

### Study Description

Study procedures for the PNC^33,34,36^ (9498 participants aged 8 to 21 years) were approved by institutional review boards of Children’s Hospital of Philadelphia and University of Pennsylvania. All participants, parents, or guardians provided informed consent, and minors provided assent. Outcomes of interest included dimensional cognitive function^37,38^ and psychopathology^39,40,41,42,43,44^ (eMethods 1 in the Supplement). Associations with these outcome measures were hypothesized for environmental factors (eMethods 2 in the Supplement)^36,45^; CNV risk scores, including measures of total size, gene content, intolerance to haploinsufficiency, and dosage sensitivity (eTables 2 to 4 and eFigures 1 and 2 in the Supplement); and 6 PGSs, including autism spectrum disorder, attention-deficit/hyperactivity disorder (ADHD), bipolar disorder, major depressive disorder (MDD), schizophrenia, and intelligence (g) (eMethods 5 in the Supplement). Owing to current genomewide association study (GWAS) limitations, PGSs could only be reliably calculated in individuals in the European ancestry cohort.^46^ The subset of PNC participants who underwent brain MRI^34,47^ were also analyzed using a measure of deviation from a normative model of brain development^48^ and its association with CNV risk scores (eMethods 6 in the Supplement). This study followed the Strengthening the Reporting of Observational Studies in Epidemiology (STROBE) reporting guideline.

### Statistical Analysis

As in prior work,^38,40,49^ cognitive and psychopathological outcomes were age-normalized prior to subsequent analyses. Biological sex and self-identified race were included as demographic covariates in statistical models, along with 10 ancestry principal components (eMethods 4 in the Supplement). Models were evaluated systematically by the stepwise inclusion of CNV risk scores, environmental stressors, and PGSs, using multivariable linear and logistic regression in the *stats* package in R version 3.6.3 (The R Foundation). All β coefficients reported were standardized to provide a measure of effect size. Using the summary.lm function in R, *t* statistics were calculated from each β estimate and its standard error, and 2-tailed *P* values indicated the probability of observing as large a *t* statistic under the null hypothesis that β = 0. Correction for multiple comparisons was performed via the Benjamini-Hochberg false discovery rate, with a threshold for statistical significance of adjusted *P* less than .05.^50^

## Results

**Figure 1.**
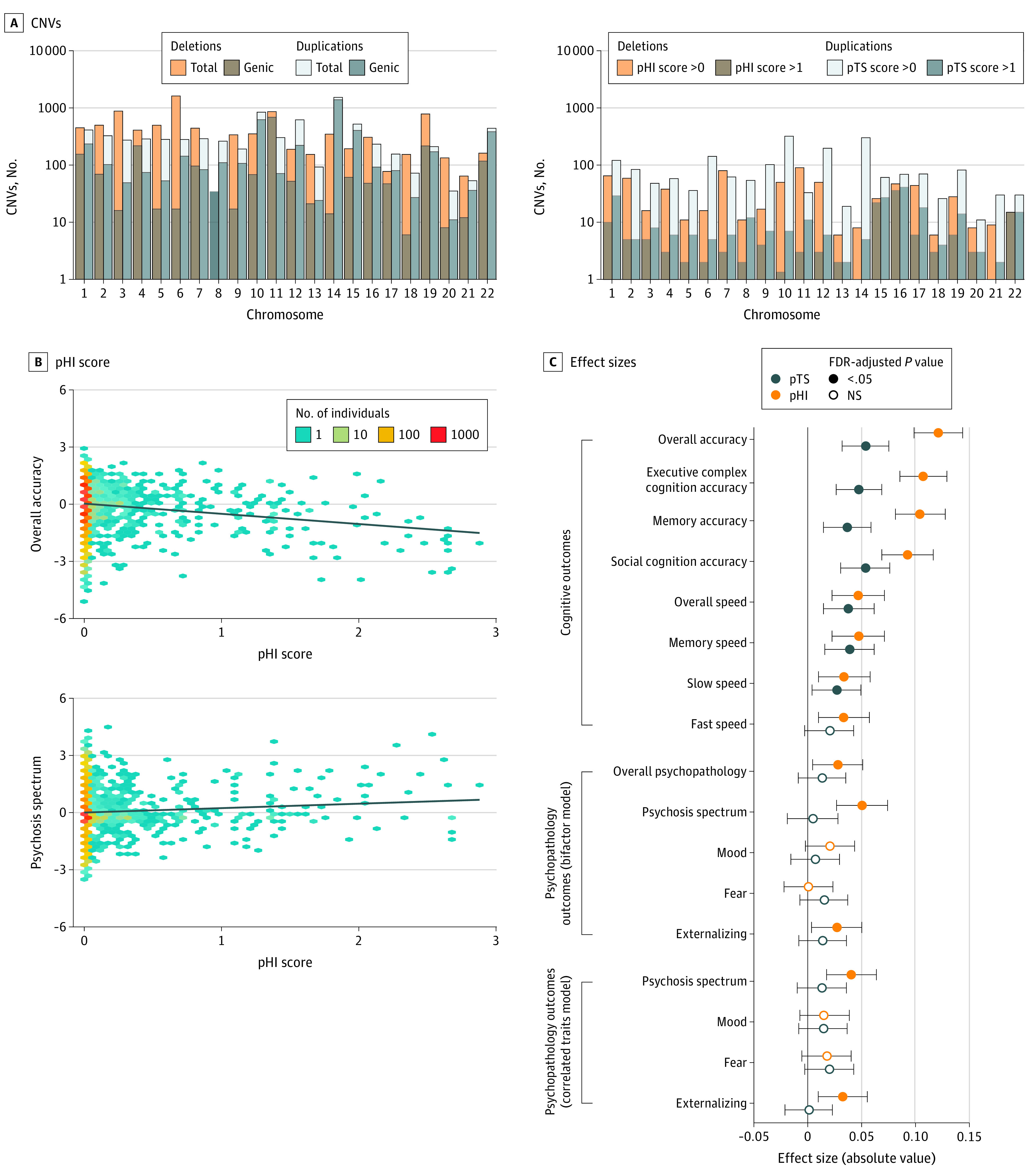
Copy Number Variants (CNVs) Larger Than 50 Kilobases Identified in the Philadelphia Neurodevelopmental Cohort and the Association of CNV Risk Scores With Cognitive and Psychopathological Outcomes A, CNVs across chromosomes. Left panel shows the total number of CNVs and the subset of genic CNVs encompassing at least 1 gene. Right panel shows the number of CNVs with risk scores greater than 0 or greater than 1. CNV risk scores were derived from the cumulative probability of haploinsufficiency (pHI; a measure of sensitivity to deletion) or probability of triplosensitivity (pTS; a measure of sensitivity to duplication). See Table 1 and eFigure 3 in the Supplement for additional information. B, Two-dimensional density plots of risk scores showing associations with overall cognition accuracy (top panel) and psychosis-spectrum symptomatology (bottom panel). C, Dot plots of effect sizes (standardized β coefficients) for associations of risk scores with 8 cognitive outcomes (top panel), and psychopathology outcomes generated via bifactor models (middle panel) and correlated traits factor models (bottom panel). Cognitive outcomes included speed and accuracy scores for specific and global measures; slow speed is summarized from items requiring deliberation, while fast speed indexes rapid decisions. All outcome measures were age-normalized. Additional covariates included self-identified race, sex, and 10 ancestry principal components. These analyses were generated based on the multiancestry sample of 7101 participants genotyped on Illumina arrays that met quality-control criteria. *P* values were corrected for 34 comparisons using Benjamini-Hochberg false discovery rate (FDR). NS indicates not significant.

Participants included 9498 youths aged 8 to 21 years; 4906 (51.7%) were female, and the mean (SD) age was 14.2 (3.7) years. The CNV sample after quality control comprised 7543 unrelated youths, 7101 genotyped on Illumina Infinium Beadchip arrays (aged 8 to 21 years; mean [SD] age, 14.2 [3.7] years; African American, 1818 [26%]; European American, 4482 [63%]; other race [including American Indian, Asian, Native Hawaiian or Other Pacific Islander, and multiracial], 801 [11%]; eTable 1 in the Supplement). In these participants, 18 185 total CNVs (10 517 deletions, 7668 duplications) were identified (Figure 1A). CNV risk scores were quantified in terms of the cumulative size of deletions or duplications; total number of genes encompassed by CNVs; intolerance scores,^18,19^ measured by genes’ probability of loss intolerance or the inverse loss-of-function observed/expected upper bound fraction (1/LOEUF); and dosage sensitivity scores,^35^ measured by the probability of haploinsufficiency (pHI) in deletions and probability of triplosensitivity (pTS) in duplications (eTables 2 to 4 and eFigures 3 and 4 in the Supplement).

### CNV Risk Score Associations With Cognition

To determine if CNVs had the hypothesized, cumulative association with cognitive outcomes, regression models were run with overall accuracy (a proxy for generalized intelligence) and multiple subdomains of cognition, showing robust associations with measures of CNV burden scores (eg, association between total CNV deletion size and overall accuracy: standardized β = −0.08; 95% CI, −0.11 to −0.06; *P* = 1.28 × 10^−13^), intolerance and dosage sensitivity scores (eg, association between CNV pHI score and overall accuracy: β = −0.12; 95% CI, −0.14 to −0.10; *P* = 7.41 × 10^−26^; Table 1). There was little evidence of specificity with respect to CNV associations with distinct cognitive domains (Figure 1B and C; eFigure 1 in the Supplement).

**Table 1.** Association of Copy Number Variant (CNV) Risk Scores With Overall Cognitive Accuracy^a^

CNV risk scores	Standardized β (95% CI)	*P* value	FDR-adjusted *P* value	Adjusted *r*^2^	AIC
pHI					
Deletion pHI	−0.121 (−0.144 to −0.099)	7.41 × 10^−26^	9.49 × 10^−24^	0.125	18 922
Duplication pTS	−0.054 (−0.076 to −0.032)	1.31 × 10^−6^	1.47 × 10^−4^		
pLI					
Deletion	−0.117 (−0.14 to −0.094)	1.03 × 10^−23^	1.30 × 10^−21^	0.124	18 928
Duplication	−0.059 (−0.081 to −0.037)	1.06 × 10^−7^	1.20 × 10^−5^		
1/LOEUF					
Deletion	−0.118 (−0.14 to −0.095)	2.94 × 10^−24^	3.73 × 10^−22^	0.123	18 937
Duplication	−0.044 (−0.066 to −0.022)	7.38 × 10^−5^	.008		
Log(pLI)					
Deletion	−0.103 (−0.126 to −0.081)	2.90 × 10^−19^	3.63 × 10^−17^	0.121	18 959
Duplication	−0.046 (−0.068 to −0.025)	3.28 × 10^−5^	.004		
N genes					
Deletion	−0.092 (−0.114 to −0.069)	1.85 × 10^−15^	2.29 × 10^−13^	0.117	18 989
Duplication	−0.022 (−0.044 to 0.000)	.049	>.99		
Total size					
Deletion	−0.084 (−0.106 to −0.062)	1.28 × 10^−13^	1.57 × 10^−11^	0.115	19 001
Duplication	0.001 (−0.021 to 0.023)	.91	>.99		
Log(1/LOEUF)					
Deletion	−0.066 (−0.089 to −0.044)	7.07 × 10^−9^	8.06 × 10^−7^	0.113	19 021
Duplication	−0.013 (−0.035 to 0.009)	.25	>.99		
pHI>0 / pTS>0					
Deletion pHI>0	−0.162 (−0.238 to −0.085)	3.24 × 10^−5^	.004	0.111	19 038
Duplication pTS>0	−0.032 (−0.084 to 0.019)	.22	>.99		

Abbreviations: AIC, Akaike information criterion; FDR, false discovery rate; LOEUF, loss-of-function observed/expected upper bound fraction; pHI, probability of haploinsufficiency; pLI, probability of loss intolerance; pTS, probability of triplosensitivity.

^a^ Rows are sorted from lowest to highest AIC, where lower AIC indicates a superior model fit. Overall accuracy scores were age-normalized, and additional covariates included self-identified race, sex, and 10 ancestry principal components. This table was generated from the multiancestry sample of 7101 participants genotyped on Illumina arrays that met quality-control criteria, and *P* values were corrected for 16 comparisons using the Benjamini-Hochberg FDR.

If CNV risk scores that incorporate annotations of intolerance and dosage sensitivity improve associations with clinically relevant outcomes, then these models should outperform simpler models based on CNV burden. This prediction was borne out, and pHI/pTS scores outperformed other annotations as measured by a decrease in Akaike information criteria (AIC) (Table 1). Because the distribution of CNV risk scores was positively skewed consistent with benign CNVs comprising the large majority (Figure 1A; eFigures 3 and 4 in the Supplement), logarithmic and categorical transformations of CNV risk scores were analyzed and also showed strong associations with outcomes (eg, log[probability of loss intolerance deletions]: β = −0.10; 95% CI, −0.13 to −0.08; *P* = 2.90 × 10^−19^; pHI greater than 0 vs pHI of 0: β = −0.16; 95% CI, −0.24 to −0.09; *P* = 3.24 × 10^−5^).

According to the multiple hit hypothesis, CNVs and environmental stressors are expected to jointly affect neurodevelopmental outcomes. Adding information about neighborhood-level socioeconomic factors and individual-level trauma exposures did strengthen associations with cognitive outcomes in addition to CNVs (eg, for overall accuracy; model with pHI/pTS and covariates: AIC = 18 922; model with environmental factors and covariates: AIC = 18 555; model with pHI/pTS and environmental measures: AIC = 18 419) (Table 2; eFigure 5 in the Supplement).

**Table 2.** Models of Cognitive and Psychopathological Outcomes Associated With Copy Number Variant (CNV) Risk Scores Indexed by Dosage Sensitivity and Environmental Factors^a^^,^^b^

Outcome	Demographic covariates		CNV risk scores		Environmental factors		Environmental factors and CNV risk scores	
	AIC	Adjusted *r*^2^	AIC	Adjusted *r*^2^	AIC	Adjusted *r*^2^	AIC	Adjusted *r*^2^
Overall accuracy	19 053	0.108	18 922	0.125	18 555	0.126	18 419	0.143
Executive complex cognition accuracy	18 752	0.148	18 646	0.161	18 291	0.166	18 181	0.179
Memory accuracy	19 499	0.031	19 416	0.043	19 087	0.039	19 002	0.051
Social cognition accuracy	19 849	0.024	19 775	0.035	19 483	0.030	19 411	0.040
Overall psychopathology	19 511	0.033	19 508	0.034	18 511	0.162	18 507	0.163
Psychosis spectrum	19 527	0.032	19 519	0.034	18 620	0.151	18 608	0.152
Externalizing	19 397	0.046	19 393	0.047	18 794	0.125	18 789	0.126
Fear	19 366	0.042	19 365	0.043	18 979	0.094	18 978	0.095
Mood	19 649	0.018	19 650	0.018	18 816	0.128	18 816	0.129

Abbreviation: AIC, Akaike information criterion.

^a^ For dosage sensitivity, probability of haploinsufficiency was used for deletions and probability of triplosensitivity was used for duplications. For environmental factors, neighborhood socioeconomic status and trauma exposures were used.

^b^ AIC and adjusted *r*^2^ are shown for models with increasing complexity, from left to right: demographic covariates only (self-identified race, sex, and 10 ancestry principal components); CNV risk scores and demographic covariates; environmental factors and demographic covariates; and CNV risk scores, environmental factors, and demographic covariates. This table was generated from the multiancestry sample of 7101 participants genotyped on Illumina arrays that met quality-control criteria. All outcome measures were age-normalized.

### CNV Risk Score Associations With Psychopathology

Compared with the association with cognitive phenotypes, CNV risk scores had subtler but significant associations with psychopathology (Table 2). Specifically, higher pHI dosage sensitivity scores were associated with higher overall psychopathology (β = 0.03; 95% CI, 0-0.05; *P* = 2.21 × 10^−2^), externalizing symptoms (β = 0.03; 95% CI, 0-0.05; *P* = 2.59 × 10^−2^) and psychosis-spectrum symptoms (β = 0.05; 95% CI, 0.03-0.08; *P* = 3.48 × 10^−5^) (Figure 1B and C). Higher pHI scores were also associated with higher odds of categorical psychiatric diagnoses (psychosis spectrum: β = 0.13; 95% CI, 0.06-0.20; *P* = 4.61 × 10^−4^; ADHD: β = 0.11; 95% CI, 0.04-0.18; *P* = .003; eTable 3 in the Supplement). CNV deletion risk scores were therefore associated with both categorical and dimensional psychopathology. In contrast to cognitive outcomes, we did not observe significant association between CNV duplication scores and psychopathology after false discovery rate correction. Similar to cognitive outcomes, adding information about environmental stressors improved models of psychopathology outcomes (eg, psychosis-spectrum symptoms; model with pHI/pTS and covariates: AIC = 19 519; model with pHI/pTS, and environmental measures: AIC = 18 608) (Table 2; eFigure 5 in the Supplement).

### Combined Analysis of CNV Risk Scores, PGSs, and Environmental Factors

**Figure 2.**
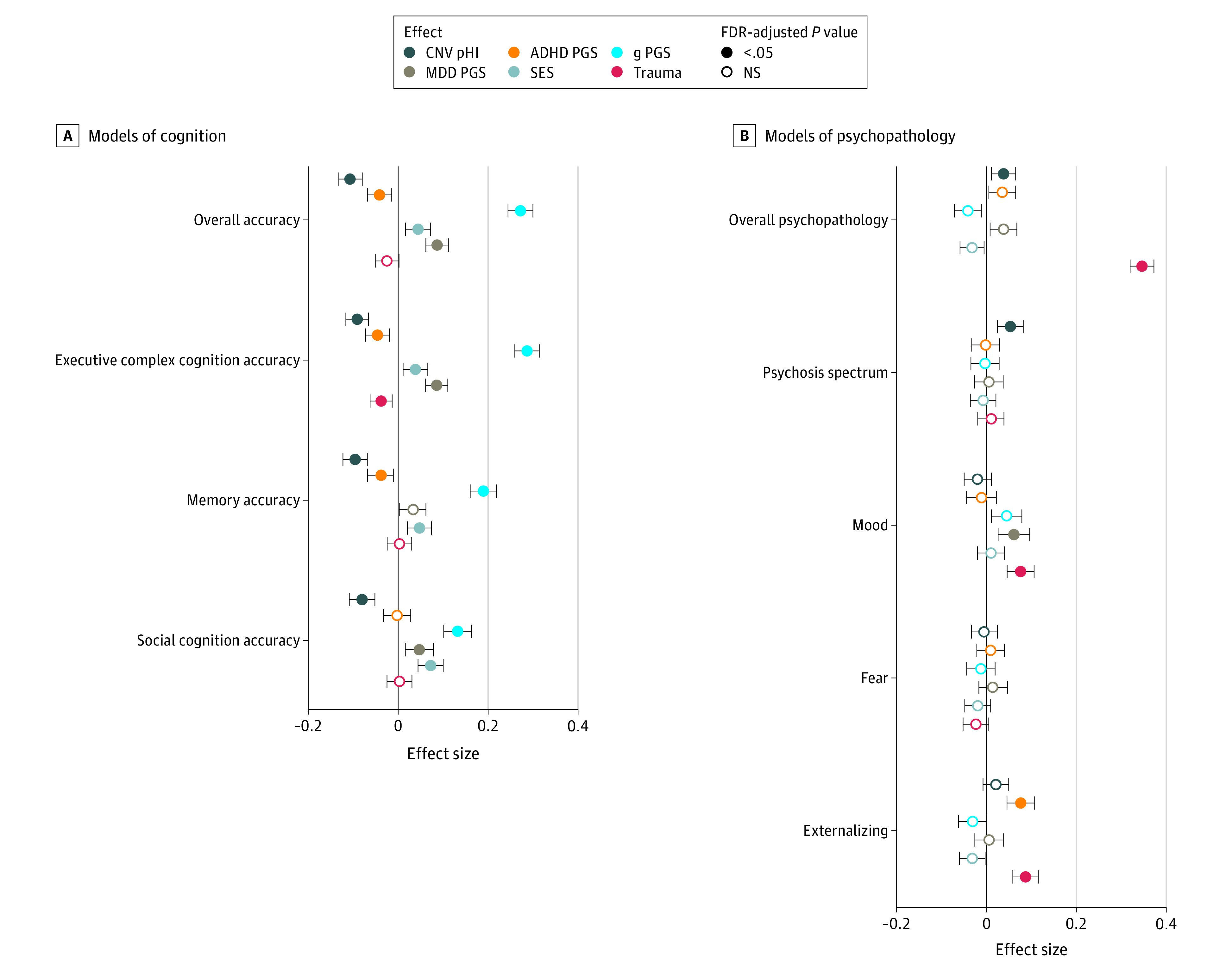
Combined Models of Developmental Outcomes and Their Joint Associations With Copy Number Variant (CNV) Scores, Environmental Factors, and Common Variant Polygenic Scores (PGSs) Points in the dot plots indicate the value of a given predictor variable’s effect size and error bars indicate 95% CIs for models of cognition (A) and psychopathology (B). For clarity, this figure shows a subset of modeled associations: CNV risk scores indexed by deletion cumulative probability of haploinsufficiency (pHI); neighborhood socioeconomic status (SES); trauma exposure; and PGSs for general intelligence (g), attention-deficit/hyperactivity disorder (ADHD), and major depressive disorder (MDD). See eFigure 6 in the Supplement for an equivalent plot showing additional associations, including CNV duplication cumulative probability of triplosensitivity and PGSs for autism spectrum disorder, bipolar disorder, and schizophrenia. All outcome measures were age-normalized, and additional covariates included self-identified race, sex, and 10 ancestry principal components in all models. This analysis was conducted in the European ancestry sample and included 4482 individuals genotyped with Illumina arrays that met quality-control criteria, and *P* values were corrected for 90 comparisons using the Benjamini-Hochberg false discovery rate (FDR). NS indicates not significant.

Owing to current GWAS limitations, we focused on the European ancestry subcohort (n = 4482) to further assess models that included PGSs in addition to environmental factors and CNV risk scores. Compared with models including only CNV risk scores and environmental factors, the addition of PGSs improved models for both cognitive and psychopathology outcomes (eTable 4 in the Supplement). By far the strongest PGS associations were between the intelligence PGS and cognition (eg, overall accuracy: β = 0.27; 95% CI, 0.24-0.30; *P* = 7.2 × 10^−78^) (Figure 2; eFigure 6 in the Supplement). Other significant associations were between the MDD PGS and mood symptoms (β = 0.06; 95% CI, 0.03-0.09; *P* = 3.58 × 10^−4^) and cognition (eg, overall accuracy: β = 0.04; 95% CI, 0.01-0.07; *P* = 3.84 × 10^−3^); between ADHD PGS and externalizing symptoms (β = 0.08; 95% CI, 0.05-0.11; *P* = 1.02 × 10^−6^) and cognition (eg, overall accuracy: β = −0.04; 95% CI, −0.07 to −0.02; *P* = 1.36 × 10^−3^). As for specific environmental factors when combined with CNVs and PGSs, the neighborhood-level factor was more strongly associated with cognition (eg, overall accuracy: β = 0.08; 95% CI, 0.06-0.11; *P* = 5.79 × 10^−11^), while trauma exposure was more strongly associated with psychopathology (eg, overall psychopathology; β = 0.35; 95% CI, 0.32-0.38; *P* = 1.1 × 10^−136^) (Figure 2; eFigure 6 in the Supplement). An exploratory analysis was conducted to test for interactions effects between CNV risk scores and environmental factors or PGSs, and no interaction effects were significant after multiple comparisons correction (eMethods 7 and eTable 5 in the Supplement).

### Neuroimaging

**Figure 3.**
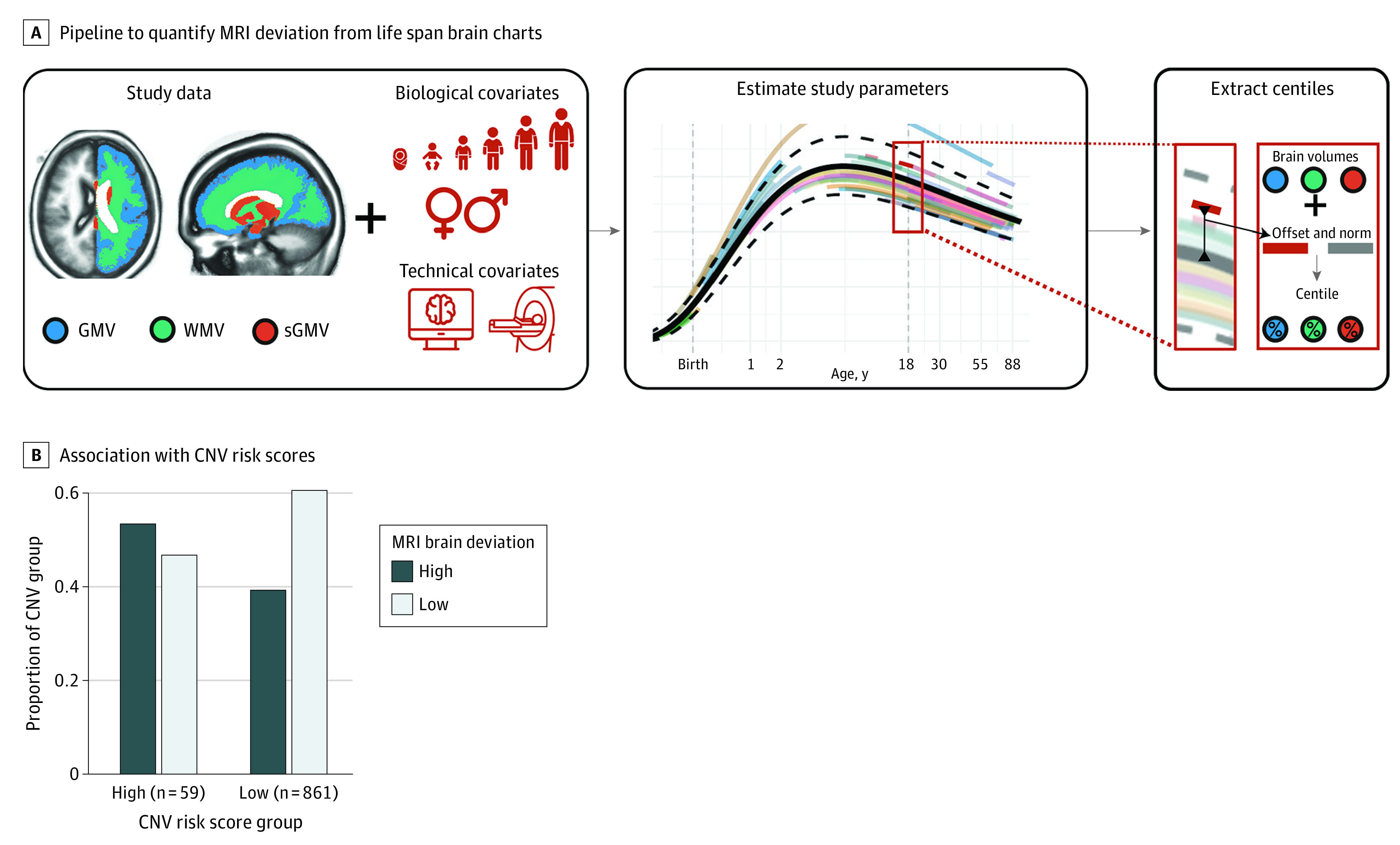
Deviations From Neuroimaging Normative Models Associated With the Presence of Copy Number Variants (CNVs) With High Risk Scores A, A schematic overview of the pipeline used for estimation of centile scores for Philadelphia Neurodevelopmental Cohort magnetic resonance imaging (MRI) data relative to a normative model. MRI data were harmonized by estimating study offset relative to other studies included in the reference sample, and centile scores were calculated for each individual based on age-specific and sex-specific expectations. Individuals are categorized as high deviation if they are in the first or tenth decile in at least 1 imaging phenotype: cortical gray matter volume (GMV), subcortical gray matter volume (sGMV), or cerebral white matter volume (WMV). B, Visualization of the comparison between the proportion of individuals with high CNV risk scores (cumulative probability of haploinsufficiency greater than 1 or cumulative probability of triplosensitivity greater than 1) categorized as high brain deviation (first or tenth decile in at least 1 imaging phenotype); individuals with high CNV risk scores categorized as low brain deviation (second to ninth decile in all imaging phenotypes); individuals with low CNV risk scores and low brain deviation; and individuals with low CNV risk scores and high brain deviation. This analysis was conducted in the subset of 920 individuals with CNV data and structural brain magnetic resonance imaging data that met quality-control criteria. eFigure 10 in the Supplement shows the full distribution of individual brain imaging–based centile scores.

High CNV risk scores were positively associated with neuroimaging deviations from normative ranges (Figure 3). Of 920 multiancestry participants with structural imaging after quality control, 59 participants were characterized as having high CNV risk scores, defined as either total pHI greater than 1 (deletions) or pTS greater than 1 (duplications). Of these participants with high-risk scores, 32 of 59 (54%) were also categorized as high deviation based on neuroimaging normative models (eMethods 6 in the Supplement) compared with 340 of 861 participants (39.5%) with lower CNV risk scores (β = 0.56; 95% CI, 0.03-1.10; *P* = .04). This result was robust to using a LOEUF-based annotation for CNV risk scores (β = 0.69; 95% CI, 0.03-1.37; *P* = .04), as well as the incorporation of a medium risk score category (high CNV risk score: β = 0.77; 95% CI, 0.11-1.46; *P* = .02; medium CNV risk score: β = 0.34; 95% CI, 0.02-0.65; *P* = .04) (eFigure 7 in the Supplement).

### Sensitivity Analyses

We performed a number of sensitivity analyses to investigate the robustness of reported findings (eMethods 8 in the Supplement). Using a literature-defined set of known pathogenic CNVs, we excluded 130 participants with known pathogenic CNVs, demonstrating that associations with CNV risk scores were not entirely due to effects of known pathogenic CNVs (although the degree of statistical significance and the strength of associations were altered for some of the outcome measures; eTables 6 and 7 and eFigure 8 in the Supplement). Further sensitivity analyses confirmed the robustness of the main results to the inclusion of X chromosome CNVs (eTable 8 in the Supplement), individuals genotyped with Affymetrix arrays (eTable 9 in the Supplement), the inclusion of demographic covariates (eTables 10 and 11 in the Supplement), and the inclusion of random effects to control for heterogeneity in array technology (eTable 12 and eFigure 9 in the Supplement).

## Discussion

The present results constitute a step toward synthesizing rare genetic, common genetic, and environmental factors to improve our understanding of their associations with clinically relevant outcomes in youth. Our study shows the joint association of CNVs (recurrent or nonrecurrent), common genetic variation (PGSs), and measures of environmental stress with clinical and subclinical psychopathology and cognitive outcomes. In general, statistical significance and effect sizes were stronger for cognitive outcomes compared with psychopathological outcomes and for deletions compared with duplications, and models were improved by the addition of information about environmental factors and PGSs as well as CNV risk scores. CNV risk scores were also associated with deviations from a normative model of MRI-derived brain structure.^51^ We show that CNV-related associations can be investigated with CNV risk scores even in cohorts not powered for genomewide discovery, which often benefit from deeper phenotyping than is typical in large-scale genetic studies.

Our results suggest that CNV risk scores are associated with a range of dimensions of psychopathology, including the psychosis spectrum. Importantly, associations with the psychosis dimension persists when controlling for overall psychopathology,^42,43,44^ suggesting an association over and above that with general psychiatric morbidity. In addition, these associations persist when known pathogenic CNVs, such as 22q11.2 deletion syndrome, are excluded from models. While the lack of significant associations with some other psychiatric symptom domains may be a false-negative owing to insufficient statistical power, the association with psychosis is consistent with an impact by both recurrent and nonrecurrent CNVs on early neurodevelopmental mechanisms that mediate risk for psychosis symptoms.^52,53,54^

It is important to note that CNVs with high risk scores, based on computational annotations of deleted or duplicated genomic segments, are not necessarily pathogenic in the sense of a known clinical association from prior literature. Relevant clinical information could be provided even for ultra-rare CNVs, where case-control studies of multiple patients with the same structural variant are not feasible but elevated risk scores have been associated with psychopathology. Moreover, even known pathogenic CNVs have variable associations with dimensional outcome measures, which can be captured by risk scores, providing information beyond that afforded by a binary index of pathogenicity. Conceptually, CNV risk scores bear similarities to PGSs, where individuals with similar PGSs do not necessarily overlap in terms of specific common variants. While PGSs can be derived for specific psychiatric outcomes based on available GWAS, however, CNV risk scores are based on CNV burden, intolerance, and dosage sensitivity of encompassed genes.

The present study compares different CNV risk scores derived from gene-level annotations of intolerance and dosage sensitivity, including recently reported models that distinguish between haploinsufficiency and triplosensitivity (eMethods 4 in the Supplement).^35^ Prior research suggests differences between haploinsufficient and triplosensitive genes in size, distance from other genes, and precision of developmental regulation.^35^ Although deletions tend to be more damaging than duplications,^22,55^ both are associated with psychiatric illness,^56^ and mechanisms of pathogenicity are likely more variable for duplications.^18,19,57,58^ An important area of future work is to continue to investigate the possibility of convergent molecular or functional pathways mediating the association between CNV risk scores and developmental outcomes,^21^ and potentially optimizing risk scores for specific psychiatric contexts.^59^

When PGSs were included in models in addition to CNV risk scores, results suggested stronger associations with cognition compared with weaker but significant associations with psychopathology dimensions, likely owing to various methodological and biological factors. Although surprising given the high-quality schizophrenia GWAS, the lack of significant correlation between schizophrenia PGS and psychotic-spectrum symptoms is consistent with prior studies, possibly reflecting that liability for adult schizophrenia generalizes poorly to subthreshold psychosis symptoms in youth.^60,61,62,63^ Studies of threshold psychotic symptoms in adults suggest that risk conferred by recurrent CNVs is augmented by high schizophrenia PGSs.^16^ Our observed associations between ADHD PGSs and externalizing symptoms, and between MDD PGSs and mood symptoms, are highly credible.^64^ The finding that MDD PGS has a positive association with overall accuracy, executive, and social cognition is surprising, but prior reports do suggest that subthreshold depressive symptoms may have positive associations with cognition (especially with social domains).^65,66,67^ Future work will continue to explore indices of common variant effects in developmental samples, where cross-disorder liability may be particularly important.^68,69^

### Limitations

Several additional methodological limitations should be noted. First, PGSs could only be reliably calculated on individuals with European ancestry. This limitation is not specific to our study but an unfortunate reality of racial bias in the underlying GWAS data that will hopefully be addressed by increasing diversity of genetic samples.^70^ Second, there was heterogeneity in the array technology used for PNC genotyping (eMethods 3, 4, 5, and 8 in the Supplement). Third, with respect to environmental stressors, temporal information about trauma was not collected, so early developmental traumatic events that may be particularly impactful could not distinguished.^71,72,73^ Genetic trios were also not available, which would have allowed for characterization of de novo variants and parent-offspring correlation in outcome measures—especially in concert with more in-depth phenotyping of parents and familial environmental exposures (eMethods 9 and eFigure 11 in the Supplement). Fourth, neuroimaging normative models were derived from global tissue volumes, where centile scores can be reliably calculated relative to a robust population-based model.^51^ A goal for future work is to incorporate additional neuroimaging phenotypes into normative models.

The results of the present study are consistent with multiple hit hypotheses about functional outcomes.^29^ CNV risk scores, PGSs, and environmental stressors, including neighborhood environment and individual-level trauma exposures (previously analyzed without incorporating genetic information^45,49^), were jointly associated with cognitive and psychopathological outcomes in a developmental sample. The lack of statistically significant interaction effects between these exposures should be interpreted cautiously, as larger samples are likely required to reliably disambiguate additive and interactive effects (eMethods 7 in the Supplement). Moreover, the clinical importance of multiple hits is supported even if only additive effects are considered.

## Conclusions

This community-based cohort study suggests that integrating multiple domains of environmental and genetic exposure, including common genetic variation indexed by PGSs and rare genetic variation indexed by CNV risk scores, may improve our understanding of contributors to psychiatric and cognitive outcomes in neurodevelopment.

## Notes

Supplement. **eMethods 1.** Cognitive and psychopathological phenotyping and factor analysis. **eMethods 2.** Quantification of environmental factors. **eMethods 3.** CNV quality control and descriptive statistics. **eMethods 4.** CNV annotation and CNV risk scores. **eMethods 5.** PGS calculation, empirical determination of ancestry, and kinship. **eMethods 6.** Neuroimaging. **eMethods 7.** Tests of CNV–PGS and CNV–environment interaction effects. **eMethods 8.** Sensitivity analyses. **eMethods 9.** Analysis of family history. **eFigure 1.** Schematic of CNV data processing pipeline. **eFigure 2.** Exclusion plot of chip frequency by number of CNV and markers. **eFigure 3.** Distribution of CNVs and CNV risk scores in the study sample. **eFigure 4.** CNV frequency generally decreases in association with increases in CNV risk score. **eFigure 5.** CNV associations with cognition and psychopathology, including environmental factors in multiancestry sample. **eFigure 6.** Combined models of developmental outcomes and their joint associations with CNV scores, environmental factors, and common variant polygenic scores (PGSs). **eFigure 7.** Brain imaging (MRI) deviation from normative models and CNV risk scores. **eFigure 8.** Sensitivity analysis excluding participants with known pathogenic CNVs. **eFigure 9.** Sensitivity analysis with random effect for Illumina Infinium Beadchip subversion. **eFigure 10.** Full distributions of individual brain imaging–based centile scores. **eFigure 11.** Combined models of developmental outcomes and their joint associations with CNV scores, environmental factors, and common variant polygenic scores. **eTable 1.** Demographic information for CNV samples after quality control. **eTable 2.** Definitions of terms related to CNV risk scores. **eTable 3.** Logistic regression models of categorical diagnoses. **eTable 4.** Models of cognitive and psychopathological outcomes. **eTable 5.** Exploratory analysis of interaction effects. **eTable 6.** Known pathogenic CNVs identified in the PNC sample. **eTable 7.** Association with CNV risk scores excluding known pathogenic CNVs. **eTable 8.** Associations with CNV risk scores including X chromosome CNVs. **eTable 9.** Associations with CNV risk scores including Affymetrix arrays. **eTable 10.** No association between pHI CNV risk scores and demographic variables. **eTable 11.** Associations without demographic covariates included in statistical models. **eTable 12. **Impact of Illumina beadchip subversion.
eReferences.

## References

[1] Girirajan S, Rosenfeld JA, Coe BP, . Phenotypic heterogeneity of genomic disorders and rare copy-number variants. N Engl J Med. 2012;367(14):1321-1331. doi:10.1056/NEJMoa1200395 22970919 PMC3494411

[2] McCarthy SE, Makarov V, Kirov G, ; Wellcome Trust Case Control Consortium. Microduplications of 16p11.2 are associated with schizophrenia. Nat Genet. 2009;41(11):1223-1227. doi:10.1038/ng.474 19855392 PMC2951180

[3] Stefansson H, Rujescu D, Cichon S, ; GROUP. Large recurrent microdeletions associated with schizophrenia. Nature. 2008;455(7210):232-236. doi:10.1038/nature07229 18668039 PMC2687075

[4] Bassett AS, Chow EW. 22q11 Deletion syndrome: a genetic subtype of schizophrenia. Biol Psychiatry. 1999;46(7):882-891. doi:10.1016/S0006-3223(99)00114-6 10509171 PMC3276595

[5] Marshall CR, Howrigan DP, Merico D, ; Psychosis Endophenotypes International Consortium; CNV and Schizophrenia Working Groups of the Psychiatric Genomics Consortium. Contribution of copy number variants to schizophrenia from a genome-wide study of 41,321 subjects. Nat Genet. 2017;49(1):27-35. doi:10.1038/ng.3725 27869829 PMC5737772

[6] Gur RE, Roalf DR, Alexander-Bloch A, McDonald-McGinn DM, Gur RC. Pathways to understanding psychosis through rare—22q11.2DS—and common variants. Curr Opin Genet Dev. 2021;68:35-40. doi:10.1016/j.gde.2021.01.007 33571729 PMC8728946

[7] Kendall KM, Rees E, Escott-Price V, . Cognitive performance among carriers of pathogenic copy number variants: analysis of 152,000 UK Biobank subjects. Biol Psychiatry. 2017;82(2):103-110. doi:10.1016/j.biopsych.2016.08.014 27773354

[8] Stefansson H, Meyer-Lindenberg A, Steinberg S, . CNVs conferring risk of autism or schizophrenia affect cognition in controls. Nature. 2014;505(7483):361-366. doi:10.1038/nature12818 24352232

[9] Kendall KM, Rees E, Bracher-Smith M, . Association of rare copy number variants with risk of depression. JAMA Psychiatry. 2019;76(8):818-825. doi:10.1001/jamapsychiatry.2019.0566 30994872 PMC6583866

[10] Wilfert AB, Sulovari A, Turner TN, Coe BP, Eichler EE. Recurrent de novo mutations in neurodevelopmental disorders: properties and clinical implications. Genome Med. 2017;9(1):101. doi:10.1186/s13073-017-0498-x 29179772 PMC5704398

[11] South ST, Lee C, Lamb AN, Higgins AW, Kearney HM; Working Group for the American College of Medical Genetics and Genomics Laboratory Quality Assurance Committee. ACMG Standards and Guidelines for constitutional cytogenomic microarray analysis, including postnatal and prenatal applications: revision 2013. Genet Med. 2013;15(11):901-909. doi:10.1038/gim.2013.129 24071793

[12] Richards CS, Bale S, Bellissimo DB, ; Molecular Subcommittee of the ACMG Laboratory Quality Assurance Committee. ACMG recommendations for standards for interpretation and reporting of sequence variations: revisions 2007. Genet Med. 2008;10(4):294-300. doi:10.1097/GIM.0b013e31816b5cae 18414213

[13] Miller DT, Adam MP, Aradhya S, . Consensus statement: chromosomal microarray is a first-tier clinical diagnostic test for individuals with developmental disabilities or congenital anomalies. Am J Hum Genet. 2010;86(5):749-764. doi:10.1016/j.ajhg.2010.04.006 20466091 PMC2869000

[14] Girirajan S, Rosenfeld JA, Cooper GM, . A recurrent 16p12.1 microdeletion supports a two-hit model for severe developmental delay. Nat Genet. 2010;42(3):203-209. doi:10.1038/ng.534 20154674 PMC2847896

[15] Dipple KM, McCabe ERB. Phenotypes of patients with “simple” mendelian disorders are complex traits: thresholds, modifiers, and systems dynamics. Am J Hum Genet. 2000;66(6):1729-1735. doi:10.1086/302938 10793008 PMC1378056

[16] Davies RW, Fiksinski AM, Breetvelt EJ, ; International 22q11.2 Brain and Behavior Consortium. Using common genetic variation to examine phenotypic expression and risk prediction in 22q11.2 deletion syndrome. Nat Med. 2020;26(12):1912-1918. doi:10.1038/s41591-020-1103-1 33169016 PMC7975627

[17] Sanislow CA, Ferrante M, Pacheco J, Rudorfer MV, Morris SE. Advancing translational research using NIMH Research Domain Criteria and computational methods. Neuron. 2019;101(5):779-782. doi:10.1016/j.neuron.2019.02.024 30844398

[18] Karczewski KJ, Francioli LC, Tiao G, ; Genome Aggregation Database Consortium. The mutational constraint spectrum quantified from variation in 141,456 humans. Nature. 2020;581(7809):434-443. doi:10.1038/s41586-020-2308-7 32461654 PMC7334197

[19] Lek M, Karczewski KJ, Minikel EV, ; Exome Aggregation Consortium. Analysis of protein-coding genetic variation in 60,706 humans. Nature. 2016;536(7616):285-291. doi:10.1038/nature19057 27535533 PMC5018207

[20] Huguet G, Schramm C, Douard E, ; IMAGEN Consortium. Measuring and estimating the effect sizes of copy number variants on general intelligence in community-based samples. JAMA Psychiatry. 2018;75(5):447-457. doi:10.1001/jamapsychiatry.2018.0039 29562078 PMC5875373

[21] Huguet G, Schramm C, Douard E, . Genome-wide analysis of gene dosage in 24,092 individuals estimates that 10,000 genes modulate cognitive ability. Mol Psychiatry. 2021;26(6):2663-2676. doi:10.1038/s41380-020-00985-z 33414497 PMC8953148

[22] Douard E, Zeribi A, Schramm C, . Effect sizes of deletions and duplications on autism risk across the genome. Am J Psychiatry. 2021;178(1):87-98. doi:10.1176/appi.ajp.2020.19080834 32911998 PMC8931740

[23] McFall ME, Mackay PW, Donovan DM. Combat-related posttraumatic stress disorder and severity of substance abuse in Vietnam veterans. J Stud Alcohol. 1992;53(4):357-363. doi:10.15288/jsa.1992.53.357 1619930

[24] Heim C, Newport DJ, Mletzko T, Miller AH, Nemeroff CB. The link between childhood trauma and depression: insights from HPA axis studies in humans. Psychoneuroendocrinology. 2008;33(6):693-710. doi:10.1016/j.psyneuen.2008.03.008 18602762

[25] van Os J, Kenis G, Rutten BPF. The environment and schizophrenia. Nature. 2010;468(7321):203-212. doi:10.1038/nature09563 21068828

[26] Martin K, McLeod E, Périard J, Rattray B, Keegan R, Pyne DB. The impact of environmental stress on cognitive performance: a systematic review. Hum Factors. 2019;61(8):1205-1246. doi:10.1177/0018720819839817 31002273

[27] McEwen BS. Stress, adaptation, and disease. allostasis and allostatic load. Ann N Y Acad Sci. 1998;840:33-44. doi:10.1111/j.1749-6632.1998.tb09546.x 9629234

[28] Gluckman PD, Hanson MA, Buklijas T, Low FM, Beedle AS. Epigenetic mechanisms that underpin metabolic and cardiovascular diseases. Nat Rev Endocrinol. 2009;5(7):401-408. doi:10.1038/nrendo.2009.102 19488075

[29] Daskalakis NP, Bagot RC, Parker KJ, Vinkers CH, de Kloet ER. The three-hit concept of vulnerability and resilience: toward understanding adaptation to early-life adversity outcome. Psychoneuroendocrinology. 2013;38(9):1858-1873. doi:10.1016/j.psyneuen.2013.06.008 23838101 PMC3773020

[30] Southwick SM, Charney DS. The science of resilience: implications for the prevention and treatment of depression. Science. 2012;338(6103):79-82. doi:10.1126/science.1222942 23042887

[31] Davies G, Lam M, Harris SE, . Study of 300,486 individuals identifies 148 independent genetic loci influencing general cognitive function. Nat Commun. 2018;9(1):2098. doi:10.1038/s41467-018-04362-x 29844566 PMC5974083

[32] Rietveld CA, Medland SE, Derringer J, ; LifeLines Cohort Study. GWAS of 126,559 individuals identifies genetic variants associated with educational attainment. Science. 2013;340(6139):1467-1471. doi:10.1126/science.1235488 23722424 PMC3751588

[33] Calkins ME, Merikangas KR, Moore TM, . The Philadelphia Neurodevelopmental Cohort: constructing a deep phenotyping collaborative. J Child Psychol Psychiatry. 2015;56(12):1356-1369. doi:10.1111/jcpp.12416 25858255 PMC4598260

[34] Satterthwaite TD, Elliott MA, Ruparel K, . Neuroimaging of the Philadelphia Neurodevelopmental Cohort. Neuroimage. 2014;86:544-553. doi:10.1016/j.neuroimage.2013.07.064 23921101 PMC3947233

[35] Collins RL, Glessner JT, Porcu E, . A cross-disorder dosage sensitivity map of the human genome. medRxiv. Preprint posted online January 28, 2021. doi:10.1101/2021.01.26.21250098 PMC9742861 35917817

[36] Moore TM, Martin IK, Gur OM, . Characterizing social environment’s association with neurocognition using census and crime data linked to the Philadelphia Neurodevelopmental Cohort. Psychol Med. 2016;46(3):599-610. doi:10.1017/S0033291715002111 26492931 PMC7263021

[37] Gur RC, Richard J, Hughett P, . A cognitive neuroscience-based computerized battery for efficient measurement of individual differences: standardization and initial construct validation. J Neurosci Methods. 2010;187(2):254-262. doi:10.1016/j.jneumeth.2009.11.017 19945485 PMC2832711

[38] Moore TM, Reise SP, Gur RE, Hakonarson H, Gur RC. Psychometric properties of the Penn Computerized Neurocognitive Battery. Neuropsychology. 2015;29(2):235-246. doi:10.1037/neu0000093 25180981 PMC4345134

[39] Kaufman J, Birmaher B, Brent D, . Schedule for Affective Disorders and Schizophrenia for School-Age Children-Present and Lifetime Version (K-SADS-PL): initial reliability and validity data. J Am Acad Child Adolesc Psychiatry. 1997;36(7):980-988. doi:10.1097/00004583-199707000-00021 9204677

[40] Calkins ME, Moore TM, Merikangas KR, . The psychosis spectrum in a young U.S. community sample: findings from the Philadelphia Neurodevelopmental Cohort. World Psychiatry. 2014;13(3):296-305. doi:10.1002/wps.20152 25273303 PMC4219071

[41] Moore TM, Calkins ME, Satterthwaite TD, . Development of a computerized adaptive screening tool for overall psychopathology (“p”). J Psychiatr Res. 2019;116:26-33. doi:10.1016/j.jpsychires.2019.05.028 31176109 PMC6649661

[42] Caspi A, Houts RM, Belsky DW, . The p factor: one general psychopathology factor in the structure of psychiatric disorders? Clin Psychol Sci. 2014;2(2):119-137. doi:10.1177/2167702613497473 25360393 PMC4209412

[43] Lahey BB, Applegate B, Hakes JK, Zald DH, Hariri AR, Rathouz PJ. Is there a general factor of prevalent psychopathology during adulthood? J Abnorm Psychol. 2012;121(4):971-977. doi:10.1037/a0028355 22845652 PMC4134439

[44] Allegrini AG, Cheesman R, Rimfeld K, . The p factor: genetic analyses support a general dimension of psychopathology in childhood and adolescence. J Child Psychol Psychiatry. 2020;61(1):30-39. doi:10.1111/jcpp.13113 31541466 PMC6906245

[45] Gur RE, Moore TM, Rosen AFG, . Burden of environmental adversity associated with psychopathology, maturation, and brain behavior parameters in youths. JAMA Psychiatry. 2019;76(9):966-975. doi:10.1001/jamapsychiatry.2019.0943 31141099 PMC6547104

[46] Schultz LM, Merikangas AK, Ruparel K, . Stability of polygenic scores across discovery genome-wide association studies. HGG Adv. 2022;3(2):100091. doi:10.1016/j.xhgg.2022.100091 35199043 PMC8841810

[47] Rosen AFG, Roalf DR, Ruparel K, . Quantitative assessment of structural image quality. Neuroimage. 2018;169:407-418. doi:10.1016/j.neuroimage.2017.12.059 29278774 PMC5856621

[48] Modenato C, Martin-Brevet S, Moreau CA, . Lessons learned from neuroimaging studies of copy number variants: a systematic review. Biol Psychiatry. 2021;90(9):596-610. doi:10.1016/j.biopsych.2021.05.028 34509290

[49] Barzilay R, Calkins ME, Moore TM, . Association between traumatic stress load, psychopathology, and cognition in the Philadelphia Neurodevelopmental Cohort. Psychol Med. 2019;49(2):325-334. doi:10.1017/S0033291718000880 29655375

[50] Benjamini Y, Hochberg Y. Controlling the false discovery rate: a practical and powerful approach to multiple testing. J R Stat Soc Series B Stat Methodol. 1995;57(1):289-300. doi:10.1111/j.2517-6161.1995.tb02031.x

[51] Bethlehem RAI, Seidlitz J, White SR, ; 3R-BRAIN; AIBL; Alzheimer’s Disease Neuroimaging Initiative; Alzheimer’s Disease Repository Without Borders Investigators; CALM Team; Cam-CAN; CCNP; COBRE; cVEDA; ENIGMA Developmental Brain Age Working Group; Developing Human Connectome Project; FinnBrain; Harvard Aging Brain Study; IMAGEN; KNE96; Mayo Clinic Study of Aging; NSPN; POND; PREVENT-AD Research Group; VETSA. Brain charts for the human lifespan. Nature. Published online April 6, 2022. doi:10.1038/s41586-022-04554-y 35388223 PMC9021021

[52] Gulsuner S, Walsh T, Watts AC, ; Consortium on the Genetics of Schizophrenia (COGS); PAARTNERS Study Group. Spatial and temporal mapping of de novo mutations in schizophrenia to a fetal prefrontal cortical network. Cell. 2013;154(3):518-529. doi:10.1016/j.cell.2013.06.049 23911319 PMC3894107

[53] Birnbaum R, Jaffe AE, Chen Q, Hyde TM, Kleinman JE, Weinberger DR. Investigation of the prenatal expression patterns of 108 schizophrenia-associated genetic loci. Biol Psychiatry. 2015;77(11):e43-e51. doi:10.1016/j.biopsych.2014.10.008 25592863

[54] Jaffe AE, Straub RE, Shin JH, ; BrainSeq Consortium. Developmental and genetic regulation of the human cortex transcriptome illuminate schizophrenia pathogenesis. Nat Neurosci. 2018;21(8):1117-1125. doi:10.1038/s41593-018-0197-y 30050107 PMC6438700

[55] Rosenfeld JA, Coe BP, Eichler EE, Cuckle H, Shaffer LG. Estimates of penetrance for recurrent pathogenic copy-number variations. Genet Med. 2013;15(6):478-481. doi:10.1038/gim.2012.164 23258348 PMC3664238

[56] Walsh T, McClellan JM, McCarthy SE, . Rare structural variants disrupt multiple genes in neurodevelopmental pathways in schizophrenia. Science. 2008;320(5875):539-543. doi:10.1126/science.1155174 18369103

[57] Hurles ME, Dermitzakis ET, Tyler-Smith C. The functional impact of structural variation in humans. Trends Genet. 2008;24(5):238-245. doi:10.1016/j.tig.2008.03.001 18378036 PMC2869026

[58] Sanders SJ, He X, Willsey AJ, ; Autism Sequencing Consortium. Insights into autism spectrum disorder genomic architecture and biology from 71 risk loci. Neuron. 2015;87(6):1215-1233. doi:10.1016/j.neuron.2015.09.016 26402605 PMC4624267

[59] Fuller ZL, Berg JJ, Mostafavi H, Sella G, Przeworski M. Measuring intolerance to mutation in human genetics. Nat Genet. 2019;51(5):772-776. doi:10.1038/s41588-019-0383-1 30962618 PMC6615471

[60] Sieradzka D, Power RA, Freeman D, . Are genetic risk factors for psychosis also associated with dimension-specific psychotic experiences in adolescence? PLoS One. 2014;9(4):e94398. doi:10.1371/journal.pone.0094398 24718684 PMC3981778

[61] Jones HJ, Heron J, Hammerton G, ; 23 and Me Research Team. Investigating the genetic architecture of general and specific psychopathology in adolescence. Transl Psychiatry. 2018;8(1):145. doi:10.1038/s41398-018-0204-9 30089819 PMC6082910

[62] Jones HJ, Stergiakouli E, Tansey KE, . Phenotypic manifestation of genetic risk for schizophrenia during adolescence in the general population. JAMA Psychiatry. 2016;73(3):221-228. doi:10.1001/jamapsychiatry.2015.3058 26818099 PMC5024747

[63] Olde Loohuis LM, Mennigen E, Ori APS, . Genetic and clinical analyses of psychosis spectrum symptoms in a large multiethnic youth cohort reveal significant link with ADHD. Transl Psychiatry. 2021;11(1):80. doi:10.1038/s41398-021-01203-2 33510130 PMC7844241

[64] American Psychiatric Association. Diagnostic and Statistical Manual of Mental Disorders. 5th ed. American Psychiatric Association; 2013.

[65] Service SK, Vargas Upegui C, Castaño Ramírez M, . Distinct and shared contributions of diagnosis and symptom domains to cognitive performance in severe mental illness in the Paisa population: a case-control study. Lancet Psychiatry. 2020;7(5):411-419. doi:10.1016/S2215-0366(20)30098-5 32353276 PMC7788266

[66] Chiappelli J, Kochunov P, DeRiso K, . Testing trait depression as a potential clinical domain in schizophrenia. Schizophr Res. 2014;159(1):243-248. doi:10.1016/j.schres.2014.08.003 25171855 PMC4177287

[67] Harkness KL, Washburn D, Theriault JE, Lee L, Sabbagh MA. Maternal history of depression is associated with enhanced theory of mind in depressed and nondepressed adult women. Psychiatry Res. 2011;189(1):91-96. doi:10.1016/j.psychres.2011.06.007 21733579

[68] Cross-Disorder Group of the Psychiatric Genomics Consortium; Cross-Disorder Group of the Psychiatric Genomics Consortium. Genomic relationships, novel loci, and pleiotropic mechanisms across eight psychiatric disorders. Cell. 2019;179(7):1469-1482.e11. doi:10.1016/j.cell.2019.11.020 31835028 PMC7077032

[69] Hughes D, Hopkinson C, Eryilmaz H, . “Children aren’t just small adults”: non-specific mapping of polygenic risk scores onto dimensional psychopathology at age 9-10 in the ABCD study. Biol Psychiatry. 2021;89(9):S223. doi:10.1016/j.biopsych.2021.02.563

[70] McGuire AL, Gabriel S, Tishkoff SA, . The road ahead in genetics and genomics. Nat Rev Genet. 2020;21(10):581-596. doi:10.1038/s41576-020-0272-6 32839576 PMC7444682

[71] Herzog JI, Schmahl C. Adverse childhood experiences and the consequences on neurobiological, psychosocial, and somatic conditions across the lifespan. Front Psychiatry. 2018;9:420. doi:10.3389/fpsyt.2018.00420 30233435 PMC6131660

[72] Dunn EC, Wang Y, Tse J, . Sensitive periods for the effect of childhood interpersonal violence on psychiatric disorder onset among adolescents. Br J Psychiatry. 2017;211(6):365-372. doi:10.1192/bjp.bp.117.208397 29097401 PMC5709674

[73] Schalinski I, Teicher MH, Nischk D, Hinderer E, Müller O, Rockstroh B. Type and timing of adverse childhood experiences differentially affect severity of PTSD, dissociative and depressive symptoms in adult inpatients. BMC Psychiatry. 2016;16(1):295. doi:10.1186/s12888-016-1004-5 27543114 PMC4992284

