# Supplementary material for: Copy Number Variant Risk Scores Associated With Cognition, Psychopathology, and Brain Structure in Youths in the Philadelphia Neurodevelopmental Cohort
Source: JAMA Psychiatry. 2022 May 11;79(7):699–709. doi: 10.1001/jamapsychiatry.2022.1017 (PMC9096695; doi:10.1001/jamapsychiatry.2022.1017)
Supplement: Supplement. — eMethods 1. Cognitive and psychopathological phenotyping and factor analysis. eMethods 2. Quantification of environmental factors. eMethods 3. CNV quality control and descriptive statistics. eMethods 4. CNV annotation and CNV risk scores. eMethods 5. PGS calculation, empirical determination of ancestry, and kinship. eMethods 6. Neuroimaging. eMethods 7. Tests of CNV–PGS and CNV–environment interaction effects. eMethods 8. Sensitivity analyses. eMethods 9. Analysis of family history. eFigure 1. Schematic of CNV data processing pipeline. eFigure 2. Exclusion plot of chip frequency by number of CNV and markers. eFigure 3. Distribution of CNVs and CNV risk scores in the study sample. eFigure 4. CNV frequency generally decreases in association with increases in CNV risk score. eFigure 5. CNV associations with cognition and psychopathology, including environmental factors in multiancestry sample. eFigure 6. Combined models of developmental outcomes and their joint associations with CNV scores, environmental factors, and common variant polygenic scores (PGSs). eFigure 7. Brain imaging (MRI) deviation from normative models and CNV risk scores. eFigure 8. Sensitivity analysis excluding participants with known pathogenic CNVs. eFigure 9. Sensitivity analysis with random effect for Illumina Infinium Beadchip subversion. eFigure 10. Full distributions of individual brain imaging–based centile scores. eFigure 11. Combined models of developmental outcomes and their joint associations with CNV scores, environmental factors, and common variant polygenic scores. eTable 1. Demographic information for CNV samples after quality control. eTable 2. Definitions of terms related to CNV risk scores. eTable 3. Logistic regression models of categorical diagnoses. eTable 4. Models of cognitive and psychopathological outcomes. eTable 5. Exploratory analysis of interaction effects. eTable 6. Known pathogenic CNVs identified in the PNC sample. eTable 7. Association with CNV risk scores excluding [file jamapsychiatry-e221017-s001.pdf]

Supplemental Online Content

Alexander-Bloch A, Huguet G, Schultz LM, et al. Copy number variant risk scores associated with cognition, psychopathology, and brain structure in youths in the Philadelphia Neurodevelopmental Cohort. *JAMA Psychiatry*. Published online May 11, 2022. doi:10.1001/jamapsychiatry.2022.1017

eMethods 1. Cognitive and psychopathological phenotyping and factor analysis
eMethods 2. Quantification of environmental factors
eMethods 3. CNV quality control and descriptive statistics
eMethods 4. CNV annotation and CNV risk scores
eMethods 5. PGS calculation, empirical determination of ancestry, and kinship
eMethods 6. Neuroimaging
eMethods 7. Tests of CNV–PGS and CNV–environment interaction effects
eMethods 8. Sensitivity analyses
eMethods 9. Analysis of family history
eFigure 1. Schematic of CNV data processing pipeline
eFigure 2. Exclusion plot of chip frequency by number of CNV and markers
eFigure 3. Distribution of CNVs and CNV risk scores in the study sample
eFigure 4. CNV frequency generally decreases in association with increases in CNV risk score
eFigure 5. CNV associations with cognition and psychopathology, including environmental factors in multiancestry sample
eFigure 6. Combined models of developmental outcomes and their joint associations with CNV scores, environmental factors, and common variant polygenic scores (PGSs)
eFigure 7. Brain imaging (MRI) deviation from normative models and CNV risk scores
eFigure 8. Sensitivity analysis excluding participants with known pathogenic CNVs
eFigure 9. Sensitivity analysis with random effect for Illumina Infinium Beadchip subversion
eFigure 10. Full distributions of individual brain imaging–based centile scores
eFigure 11. Combined models of developmental outcomes and their joint associations with CNV scores, environmental factors, and common variant polygenic scores
eTable 1. Demographic information for CNV samples after quality control
eTable 2. Definitions of terms related to CNV risk scores
eTable 3. Logistic regression models of categorical diagnoses
eTable 4. Models of cognitive and psychopathological outcomes
eTable 5. Exploratory analysis of interaction effects
eTable 6. Known pathogenic CNVs identified in the PNC sample
eTable 7. Association with CNV risk scores excluding known pathogenic CNVs
eTable 8. Associations with CNV risk scores including X chromosome CNVs
eTable 9. Associations with CNV risk scores including Affymetrix arrays
eTable 10. No association between pHI CNV risk scores and demographic variables

© 2022 Alexander-Bloch A et al. *JAMA Psychiatry*.

eTable 11. Associations without demographic covariates included in statistical models
eTable 12. Impact of Illumina beadchip subversion
eReferences.

This supplementary material has been provided by the authors to give readers additional information about their work.

© 2022 Alexander-Bloch A et al. *JAMA Psychiatry*.

eMethods 1. Cognitive and psychopathological phenotyping and factor analysis

Recruitment, cognitive and clinical phenotyping in the PNC have been described previously[1–3]. Briefly, 9,498 individuals (8-21 years) were recruited from the Children's Hospital of Philadelphia (CHOP) care network. All participants, or their parents or guardians provided informed consent, and minors provided assent. Study procedures were approved by IRBs of CHOP and University of Pennsylvania

PNC participants were administered the Penn computerized neurocognitive battery (CNB), designed to measure domains of cognition that have been empirically linked to brain systems [4]. Using an exploratory and confirmatory factor analytic approach detailed previously [3], individuals were scored on dimensions of "Executive-Complex Cognition", "Memory", and "Social cognition" accuracy – as well as measures of cognitive speed. Overall accuracy and speed were also estimated with unidimensional factor models using least-squares extraction. Prior work suggests that a combined Executive-Complex Cognition accuracy factor may be a good proxy for IQ, while overall accuracy may be a good proxy for 'general intelligence' (g-factor) [3]. For consistency with prior work, all cognitive outcomes were age-normalized, using the residuals of a linear model with age as the predictor variable, and z-scored prior to further analysis.

Participants were also administered the GOASSESS, a comprehensive computerized tool for structured evaluation of psychopathology domains based on the Kiddie Schedule for Affective Disorders and Schizophrenia (K-SADS) [1,5]. From GOASSESS items, overall psychopathology was calculated using a bifactor model [6]. This single continuous measure, sometimes referred to as the p-factor [7,8], represents liability to overall psychopathology in youths [9]. Other domains of psychopathology were "Psychosis-Spectrum", "Externalizing", "Fear (phobias)" and "Mood-Anxiety" factors. We also report results from a correlated traits model, which includes the same specific symptom domains without extracting an orthogonal measure of overall psychopathology [6]. Categorical determinations were also made using the GOASSESS to identify individuals meeting criteria for ADHD, depression, anxiety, or psychosis spectrum [1,10]. For consistency with prior work, all psychopathology outcomes were age-normalized, using the residuals of a linear model with age as the predictor variable, and z-scored prior to further analysis.

As previously reported[1,3,6], cognitive and psychopathological measures correlate non-trivially. Our factor analytic approach was designed to capture cognitive and behavioral domains supported by psychological theory and prior literature, rather than to constrain factors to be orthogonal. We note that it is common to investigate multiple cognitive or psychopathological outcome measures that are not expected to be independent of one another, which reflects the reality of high correlation among domains of cognitive function as well as psychiatric co-morbidity [11]. False Discovery Rate (FDR) correction for multiple comparisons across multiple outcome measures that are not independent is expected to be conservative, i.e., this procedure is not expected to inflate the false discovery rate [12]. However, it is important to take these points into consideration when interpreting results; it is not justified to infer that reported associations with different cognitive and psychopathological outcomes are necessarily independent. As discussed above, an exception is the bifactor model of psychopathology domains, which models psychopathology factors orthogonally by accounting for an overall psychopathology factor that "absorbs" the inter-factor correlations. Thus for example, the overall psychopathology factor is orthogonal to the psychosis factor estimated from the bifactor model. This additional step is taken to justify the inference that, for example, an effect on psychosis is not attributable to an

© 2022 Alexander-Bloch A et al. *JAMA Psychiatry*.

effect on overall psychopathology. Note that while a bifactor model of neurocognition was estimated (see REF[3]), scores were not generated from this model due to poor determinacy [13]. The neurocognitive scores are therefore limited to the correlated-traits model, in which they were well-determined.

© 2022 Alexander-Bloch A et al. *JAMA Psychiatry*.

eMethods 2. Quantification of environmental factors

Environmental variables were quantified from two principal sources. First, an exploratory factor analysis identified a cumulative socioeconomic environment factor for each individual based on neighborhood-level data (including income levels, education levels, percent female residents, etc.) as previously described [14]. Second, an individual-level measure of trauma exposure was derived as a total count of traumatic experiences from a list of nine items included in the GOASSESS, including experiencing a natural disaster or bad accident, witnessing another person killed or badly beaten, witnessing a dead body, being concerned that a close contact was badly injured or killed, and experiencing assault [1]. See eMethods 9 for a supplemental analysis of psychiatric history and its relationship with environmental factors discussed above.

© 2022 Alexander-Bloch A et al. *JAMA Psychiatry*.

eMethods 3. CNV quality control and descriptive statistics

CNV processing steps are illustrated in eFigure 1. We applied a series of quality control procedures to CNV output that have been used previously in similar published work [15,16]. Code is available at https://github.com/BGDlab/cnv-study and https://github.com/MartineauJeanLouis/MIND-GENESPARALLELCNV. We inspected chip-level attributes and determined if they met stringent quality cutoffs with respect to call rate (≥95%) log r ratio standard deviation (<0.35), B allele standard deviation (<0.08) and absolute value of wave factor (<0.05). All probe coordinates were updated from hg18 to hg19.

CNVs were called using the QuantiSNP[17] and PennCNV[18] algorithms. Both algorithms are freely available and detect CNVs using hidden Markov models (HMMs) of SNP genotype data, and have been validated for Illumina and Affymetrix arrays[19]. Implemented in MATLAB, QuantiSNP uses an objective Bayes approach to detect CNVs based on the LogR ratio and the B allele frequency for each SNP marker[17]. Implemented in PERL and C, PennCNV infers CNVs based on multiple sources of information including the log r ratio and B allele frequency at each SNP marker, the distance between neighboring SNPs, and an adjustment for "genomic waves" using a regression model GC content [20]. To minimize false discoveries, the overlap between CNVs detected from each algorithm was detected using CNVision [21], and only CNVs with 70% overlap between the algorithms were included in subsequent analysis.

To minimize false discoveries, we also implemented a series of additional series of filtering steps. Following REF[16], we initially excluded arrays with a suspiciously high number of CNVs detected (defined as ≥50 for low resolution arrays with <1 million probes, and ≥200 for high resolution arrays with ≥1 million probes) (see eFigure 2). CNVs were then filtered to ensure they were greater than 50,000 base pairs in size; they had a confidence score rating ≥ 30 from either QuantiSNP or PennCNV; they did not have >50% overlap with segmental duplications, the major histocompatibility complex (MHC), centromeric regions or telomeric regions. Finally, chips were excluded if, after CNV filtering, they had >30 CNVs or >8Mb total CNV burden. After exclusion criteria based on chip quality control and genetic relatedness within the sample (with a threshold of third degree relatives), information about CNVs was available for N=7,543 participants.

It is important to note that the PNC was genotyped in 15 batches, using ten different types of Affymetrix and Illumina arrays by the CHOP Center for Applied Genomics. Arrays have different probe coverage of given genomic regions, which has the potential to impact CNV detection sensitivity/specificity; this is the principal reason we limited our analysis large CNVs >50,000 bps which are more reliably detected across platforms. We elected to use all available probes in CNV detection to maximize discovery potential. To assess the impact of differences in array technology, main analyses were conducted using subjects genotyped with Illumina arrays (N=7,126, all genotyped with Illumina Infinium Beadchip subversions) with additional subjects included in sensitivity analyses (eMethods 8). We also conducted analyses where Illumina Chip Version was modelled as a random effect in statistical models of the association with outcome variables, as well as selected analyses within Chip Version, which showed convergent associations with outcomes (eMethods 8).

© 2022 Alexander-Bloch A et al. *JAMA Psychiatry*.

For families with more than one participant in the PNC, determined via kinship analysis in KING, we chose a single random family member to be included in subsequent analyses. For individuals with more than one chip, we selected the highest quality chip by log r standard deviation to be included in subsequent analyses. While systematic re-genotyping to validate CNVs calls was not possible for this study because sample aliquots for the PNC are largely exhausted, a subset of 1,515 participants had been genotyped more than once on different chips by the CHOP Center for Applied Genomics. For these participants, we compared the highest quality chip by log r standard deviation (which was used in all subsequent analyses) with the second highest quality chip by log r standard deviation, in terms of the reliability of the CNV risk scores as indexed by pHI (Pearson's r=0.83, t=58.018, df=1,513, p=4.44e-53). This suggests high reliability for CNV risk scores that are the basis of the results reported in this paper.

After the full quality control procedure, descriptive statistics were calculated at the sample (array) level and the CNV level. At the array level, descriptive statistics were for log r ratio standard deviation (median 0.15; mean 0.16; range 0.05-0.34; standard deviation, 0.04), B allele frequency standard deviation (median 0.039; mean 0.039; range 0.016-0.074; standard deviation, 0.009), absolute value of wave factor (median 0.01; mean 0.01; range 0.004-0.04; standard deviation, 0.005), and the total number of CNVs (median 2; mean 2.55; range 0-11; standard deviation, 1.96).

Descriptive statistics at the CNV level – across 18,185 CNVs (10,517 deletions, 7,668 duplications) identified in the sample after quality control – were calculated for number of SNPs (median 27; mean 44.99; range 3- 5192; standard deviation, 84.25), length in Mb (median 0.13; mean 0.21; range 0.05-6.84; standard deviation, 0.28), confidence score (median 68.25; mean 117.91; range 30.0-17611.20; standard deviation, 229.38), and percent overlap between CNVs called by the two algorithms, PennCNV and QuantiSNP (median 100%; mean 95.4%; range 70-100%; standard deviation, 8.0%). The distribution of CNVs in terms of size and chromosomal location are shown in eFigure 3A-B. Approximately 36% of CNVs contained at least one gene. Using the Database of Genomic Variants (DGV, http://dgv.tcag.ca) "gold standard" as a reference for population frequency, 42% of PNC CNVs have a population frequency >0.01, 24% of PNC CNVs have a population frequency >0.001 and ≤0.01, 16% of PNC CNVs have a population frequency >0.0001 and ≤0.001, and 18% of PNC CNVs have a population frequency ≤0.0001.

© 2022 Alexander-Bloch A et al. *JAMA Psychiatry*.

eMethods 4. CNV annotation and CNV risk scores

As defined in eTable 2, with respect to terminology we use the phrase "CNV risk score" when referring to quantitative measures of predicted risk based on annotating individual's CNVs. More specifically, we use the term CNV burden when referring to annotations of total size or number of genes encompassed by CNV, the term intolerance when referring to annotations based on intolerance to loss of function (i.e. pLI and LOEUF scores), and the term dosage sensitivity when referring to annotations based on deletion or duplication sensitivity (i.e., pHI and pTS). We use the phrase "known pathogenic" CNVs when referring to CNVs with known associations with cognition or psychopathology from prior literature.

We annotated CNVs using Gencode V19 (hg19) with ENSEMBL (https://grch37.ensembl.org/index.html) to derive CNV risk scores. Specifically, CNVs were scored on multiple measures of CNV burden, including total size (number of base pairs) and the total number of genes (the number of genes fully encompassed by the CNV) (eFigure 3C). Two measures of CNV intolerance were also calculated (eFigure 3D). 1) The probability of loss intolerance (pLI) is the probability that a gene is intolerant to a loss of function mutation, ranging from 0-1 with a relatively bimodal distribution. For a CNV, the cumulative pLI was calculated as the sum of pLI of the genes encompassed by the CNV. 2) The loss of function observed/expected upper bound fraction (LOEUF) is a continuous measure ranging from 0 to 2, where values below 0.35 are typically suggestive of intolerance. For a CNV, the cumulative inverse LOEUF (i.e., 1/LOEUF) was calculated as the sum of the inverse LOEUF of the genes encompassed by the CNV [16]. Finally, measures of dosage sensitivity were calculated as the probability of haploinsuffiency (pHI) and probability of triplosensitivity (pTS), which quantify the probability (ranging from 0-1) that a gene is sensitive to copy number loss or copy number gain, respectively (eFigure 3E). The pHI and pTS used in the current analyses were derived from a previously reported statistical model based on 753,994 genomes, which mapped dosage sensitivity for all genes across the genome [22]. In contrast to pHI and LOEUF which were used to annotate both deletions and duplications (although the impact of deletions and duplication was modelled separately), pHI was used to annotate deleted genes while pTS was used to annotate duplicated genes. For a CNV deletion, the cumulative pHI was calculated as the sum of the pHI of genes encompassed by the CNV; for a CNV duplication, the cumulative pTS was calculated as the sum of the pTS of genes encompassed by the CNV. Participant-level CNV risk scores were summed across all of a participant's CNVs, with deletions and duplications considered separately.

For CNV deletions, overall results at the participant level were for total size in Mb (median 0.1; mean 0.2, range 0-6.8; standard deviation, 0.3), number of fully encompassed genes (median 0; mean 0.831 range 0-51; standard deviation, 2.7), cumulative 1/LOEUF (median 0; mean 0.68; range 0-78.6; standard deviation, 3.6), pLI (median, 0; mean, 0.1; range, 0-12.3; standard deviation, 0.54), and pHI (median 0; mean, 0.1; range 0-16.8; standard deviation, 0.70). For CNV duplications, overall results at the participant level were for total size in Mb (median 0.1; mean 0.3, range 0-7.6; standard deviation, 0.5), number of fully encompassed genes (median 0; mean 1.6 range 0-60; standard deviation, 3.6), cumulative 1/LOEUF (median 0; mean 1.3 range 0-97.7; standard deviation, 3.7), pLI (median 0; mean 0.1 range 0-16.9; standard deviation, 0.54), and pTS (median 0; mean 0.2 range 0-23.8; standard deviation, 0.92). eFigure 3C-E show the distribution across the sample of these CNV risk scores. Note that the interpretation of

© 2022 Alexander-Bloch A et al. *JAMA Psychiatry*.

individuals with cumulative pHI, 1/LOEUF, pTS or pHI close to zero is that these individuals are expected not to have damaging CNVs, based on the models of CNV intolerance and dosage sensitivity described above. A score of zero could arise if a participant has no CNVs, if none of the CNVs encompass genes, or if none of the encompassed genes is intolerant or dosage sensitive according the respective annotation scheme. As expected, there was a clear negative relationship between CNV in-sample frequency and CNV risk scores (eFigure 4). There were no significant associations between CNV risk scores (e.g. indexed by pHI) and biological sex, race, or age (eTable 10).

Following prior analyses [15,16], we identified known pathogenic CNVs in the sample based on 40% overlap with CNVs from a comprehensive systematic literature review [23–27]. In total, 130 total participants with 38 distinct known pathogenic CNVs were identified (see eTable 6). Participants with these CNVs were also excluded in a sensitivity analysis (eMethods 8).

As previously reported, pHI and pTS are generally highly correlated per gene (Pearson's r=0.69), though exceptions certainly occur. As would be expected due to the cumulative effect of multiple genes also being related to CNV size, the correlation is even higher when considering cumulative pHI and pTS within large CNVs – e.g. within 118 known pathogenic autosomal CNVs [23–27] (Pearson's r=0.93). Note that simply comparing pHI scores with pTS scores across CNVs in terms of their numeric values does not provide a meaningful indicator of predicted risk. While both pHI and pTS range from 0-1, numeric equivalence does not imply equal predicted risk. For example in REF [22], different thresholds were used to establish genes that were categorically haploinsufficient (pHI>0.84) and triplosensitive (pTS > 0.993) with comparable effect sizes. This is one reason why, in terms of their effect on outcome measures, deletions and duplications were always modelled with separate terms in our statistical models. As described in the main text, and consistent with the fact that deletions are typically more damaging than duplications, the effect size of deletions was in general markedly higher than that of duplications across outcome measures.

Consistent with the fact that CNVs with large effect sizes on developmental outcomes are rare events, CNV risk scores are positively skewed (eFigures 3-4). It is important to note that modelling assumptions were not violated by skewness in an independent variable[30], and because of this skewness we also explored categorical and logarithmic transformations of CNV scores. Our main results were convergent when using these nonlinear transformations. Moreover, model fits as assessed by AIC were superior for untransformed CNV scores (e.g., see Table 1), which are also consistent with prior related work [15,16]. We note that regression approaches designed to model rare events, such as Poisson regression and related approaches, would be applicable if modelling CNV scores as the outcome of interest (rather than a predictor variable)[31]. It will be instructive to further explore the impact of model assumptions on CNV associations in future work.

© 2022 Alexander-Bloch A et al. *JAMA Psychiatry*.

eMethods 5. PGS calculation, empirical determination of ancestry, and kinship

The approach to derive polygenic scores (PGSs) in the PNC has recently been described in detail [32]. Briefly, prior to imputation, PLINK 1.9 [33] was used to remove single nucleotide polymorphisms (SNPs) with > 5% missingness, samples with more than 10% missingness, and samples in which genotyped sex was different from reported sex. Genotypes were phased (Eagle v.2.4) and imputed to the 1000 Genomes Project Other/Mixed GRCh37/hg19 reference panel (Phase 3 v.5) using Minimac 4 via the Michigan Imputation Server [45]. Following imputation, only polymorphic sites with imputation quality R2 ≥ 0.7 and MAF ≥ 0.01 were retained. Multi-dimensional scaling (MDS) of the imputed genotypes was conducted using KING v.2.2.4 [34] to identify the top ten ancestry principal components (PCs) for each sample. These PCs were projected onto the 1000 Genomes Project PC space, and genetic ancestry was inferred using the e1071 [35] support vector machines package in R. Based on these inferences, a European ancestry cohort was established and other ancestry groups were excluded from PGS analyses based on strong evidence of European bias in existing GWAS and PGS calculations [32]. A second round of unprojected MDS was then performed within the European ancestry group to produce ten PCs that were included as additional covariates in statistical models within the European ancestry cohort. KING was also used to identify all pairwise relationships out to third degree relatives based on estimated kinship coefficients and inferred IBD segments, and a single random family member was selected for subsequent analyses.

PRS-CS (PRS using SNP effect sizes under continuous shrinkage) [36] was used to infer posterior effect sizes of the SNPs based on specific GWAS summary statistics. Like other Bayesian-based techniques such as LDpred[37] and SBayesR[38], PRS-CS has the substantial advantage of not relying on processes of linkage disequilibrium (LD) clumping and p-value thresholding[36] and these approaches have been shown to yield more predictive PGSs than those produced using older methodologies[39]. To ensure convergence of the underlying Gibbs sampler algorithm, we ran 25,000 Markov chain Monte Carlo (MCMC) iterations and designated the first 10,000 MCMC iterations as burn-in. The PRS-CS global shrinkage parameter was set to 0.01 when the discovery GWAS had a SNP sample size that was less than 200,000; otherwise, it was learned from the data using a fully Bayesian approach. Default settings were used for all other PRS-CS parameters. Raw PGS were produced from the posterior effects using PLINK 1.9, and PGSs were standardized using a z-transformation for use in all subsequent analyses.

GWAS summary statistics for specific disorders were based on six GWAS, for attention deficit hyperactivity disorder (ADHD)[40]; autism spectrum disorder (ASD) [41]; major depressive disorder (MDD) [42]; bipolar disorder (BPD)[43]; schizophrenia (SCZ) PGC wave 3 [44,45]; and intelligence[46]. Sample sizes of the underlying GWAS referenced above were as follows for ADHD (n=20,183 cases, 35,191 controls), ASD (n=18,381 cases and 27,969 controls), MDD (n=246,363 cases and 561,190 controls), BPD (n=20,352 cases and 31,358 controls), SCZ (n= 69,369 cases and 236,642 controls), and intelligence ($n = 269{,}867$). The estimated proportion of variance due to common variants based on the above GWAS (h^2_{SNP}) were as follows for ADHD (h^2_{SNP}=0.22), ASD (h^2_{SNP}=0.12), MDD (h^2_{SNP}=0.089), BPD (h^2_{SNP}=0.17–0.23 depending on assumed prevalence [0.5-2.0%)]), SCZ (h^2_{SNP}=0.24), and intelligence (h^2_{SNP}=0.19). Note that for ADHD, we based our PGS on GWAS excluding participants from CHOP (who may have overlapped with the PNC)[40].

© 2022 Alexander-Bloch A et al. *JAMA Psychiatry*.

The inclusion of ancestry PCs in statistical models was supported by their modest and significant associations with cognitive and psychopathology outcome variables. When included in a regression model as the effects of interest, in models of 4 cognitive outcomes and 5 psychopathology outcomes, effect sizes (standardized beta coefficients) ranged from 0.00-0.08 (median, 0.01). The association was significant (FDR-adjusted $P<0.05$, 90 multiple comparisons) for 10 associations: PC1 and overall accuracy (regression $\beta=-0.08$, $p=2.35e-08$), executive-complex accuracy (regression $\beta=-0.7$, $p=1.41e-07$), social cognition accuracy (regression $\beta=-0.05$, $p=1.68e-03$), and memory accuracy (regression $\beta=-0.07$, $p=4.36e-07$); PC4 and overall accuracy (regression $\beta=0.03$, $p=8.04e-03$), executive-complex accuracy (regression $\beta=0.03$, $p=1.03e-02$), and memory accuracy (regression $\beta=0.03$, $p=1.37e-02$); PC5 and overall accuracy (regression $\beta=0.04$, $p=6.8e-03$) and executive-complex accuracy (regression $\beta=0.04$, $p=3.00e-03$); and PC9 and memory accuracy (regression $\beta=0.04$, $p=0.05e-03$). There were no statistically significant associations between the genetic ancestry PCs and psychopathology outcomes, specifically.

© 2022 Alexander-Bloch A et al. *JAMA Psychiatry*.

eMethods 6. Neuroimaging

Structural MRIs were acquired on a single 3T Siemens TIM Trio scanner 32-channel head coil as described previously [2], using a T1-weighted magnetization-prepared, rapid-acquisition gradient-echo (MPRAGE) sequences (TR = 1810ms; TE = 3.51 ms; FoV = 180 × 240 mm; resolution 0.9375 × 0.9375 × 1 mm). All scans were manually rated for image quality as described previously [47], and poor quality scans were excluded from downstream analyses. Scans were processed in FreeSurfer v5.3.

Given prior evidence of an association between pathogenic CNVs and alterations in brain size[48], and well-replicated associations between brain size and intelligence [49] and schizophrenia detectable with MRI [50], we predicted that CNVs would impact volumetric structural brain MRI phenotypes in the PNC. The wide range of neuroanatomical alterations reported by structural neuroimaging studies of specific CNVs, however, prohibited a targeted neuroanatomical hypothesis [48] as well as a unique hypothesis about the directionality of alterations. Indeed, pathogenic CNVs are known to cause increases or decreases (depending on the specific variant) in total brain volume and regional brain structures [49–52]. We therefore explored a hypothesis based on deviation from normative developmental expectations in brain size, in any direction and across multiple imaging phenotypes. Using a recently described approach [55], we deployed a normative model of brain development to quantify the imaging phenotypes of individual PNC participants relative to age- and sex-normalized growth charts. Cortical gray matter volume (GMV), subcortical gray matter volume (sGMV) and white matter volume (WMV) were classified as either within the normative range (10%-90%), infranormal (<10%) or supranormal (>90%). Categorically, individuals were classified as 'low deviation' if they were within the normative range for all phenotypes, and 'high deviation' if they were either infranormal or supranormal in any phenotype. The neuroimaging analysis focused on this omnibus test of a deviation from normative trajectories in order to maximize statistical power given the relatively smaller number of participants in the neuroimaging sample. The rationale for this procedure was also based also on its similarity to recent work showing categorical deviations from normative ranges in schizophrenia[56].

Statistical tests were performed using logistic regression with imaging deviation as the outcome variable and with CNV-status as the predictor variable of interest. Covariates included ancestry, SES, trauma exposure, and a manual rating of image quality[47]. As normative imaging models were stratified for sex and age, these were not included as covariates in baseline models; however, the neuroimaging results reported in the main text and below were unchanged when including sex and age as additional covariates as well as for models without covariates included.

To supplement the results describing neuroimaging deviation associated with CNV risk scores derived from pHI and pTS as presented in the main text (Figure 3), we replicated the analysis using LOEUF-based CNV risk scores. After genetic and imaging quality control, there were 920 multi-ancestry participants with structural imaging. Of this cohort, 38 participants were characterized as having high CNV risk scores, defined as total deletion 1/LOEUF score > 2.94 (corresponding to LOEUF < 0.34, which has been suggested as the threshold for loss-of-function intolerance at the single gene level[16,28]). Of these participants with high CNV risk scores, 58% were also categorized as high deviation from neuroimaging normative models, compared to only 40% of participants with low CNV risk scores (logistic regression β=0.68, p=0.047) (see eFigure 7A). We performed an additional supplemental analysis where we re-characterized CNV risk

© 2022 Alexander-Bloch A et al. *JAMA Psychiatry*.

scores to incorporate a category of medium CNV risk scores defined as 0 < total 1/LOEUF score ≤ 2.94, which comprised 208 participants. The low CNV risk score group then comprised remaining participants with total 1/LOEUF score = 0. This stratification resulted in an association with neuroimaging-based brain deviation as follows: low CNV risk score group, 38% high neuroimaging deviation; medium CNV risk score group, 46% high neuroimaging deviation; high CNV risk score group, 58% high neuroimaging deviation (high risk score group, β=0.77, p=0.024; medium risk score group, β=0.34, p=0.036; eFigure 7B). Note that these β coefficients represent the effect on the log-odds ratio compared to the low CNV risk score reference group. See eFigure 10 for plots of the full distributions of the neuroimaging centile scores.

In sum, although the overall power to detect neuroimaging alterations are low because of the greatly decreased sample size in the neuroimaging cohort compared the whole PNC, results support the hypothesis of increased brain deviation from normative ranges associated with elevated CNV risk scores.

© 2022 Alexander-Bloch A et al. *JAMA Psychiatry*.

eMethods 7. Tests of CNV–PGS and CNV–environment interaction effects

It is of considerable theoretical and clinical interest whether CNV risk scores have additive or interactive effects with both common variant PGSs as well as environmental factors. However, compared to modelling additive effects, statistical power to reliably distinguish interaction effects is likely to be substantially lower [57], a problem which may be compounded when considering relatively rare events such as the presence of CNVs with high risk scores. Without a clear rationale for targeted testing of specific interactions, we performed an exploratory analysis where we systematically tested for interactions between CNV risk scores (indexed by pHI and pTS) and environmental factors (SES and trauma exposures), and between CNV risk scores and the six PGSs queried in main analyses (ASD, ADHD, SCZ, BPD, MDD, and intelligence) for four cognitive outcomes (overall accuracy, executive complex cognition accuracy, social cognition accuracy, memory accuracy) and five psychopathological outcomes (mood, fear, externalizing, psychosis spectrum, and overall psychopathology from bifactor model described above). Not surprisingly given the combinatorial multiple testing burden (144 separate interaction effects), none of these effects was significant after FDR-correction for multiple comparisons. eTable 5 shows exploratory results (uncorrected $p<0.05$). We conclude that reliably distinguishing interactive effects will require larger sample sizes or samples enriched for genetic mutations – or alternatively, targeted testing of a smaller number of *a priori* specified hypotheses. This remains an important area for future work.

eMethods 8. Sensitivity analyses

As described in eMethods 4, we identified 130 total participants with 38 known pathogenic CNVs in the sample, and we excluded these participants in sensitivity analyses in order to ascertain whether main results were due entirely to the contribution of known pathogenic CNVs. eTable 6 provides a list of known pathogenic CNVs identified as well as the cumulative dosage sensitivity scores (pHI and pTS) associated with these CNVs. As shown in eTable 7 and eFigure 8, main results – including significant associations between CNV risk scores and cognitive and psychopathological outcomes – were in general robust to this sensitivity analysis. Specifically, the associations between overall accuracy and high-risk-score deletions indexed by pHI (β=-0.049, p=8.853e-06) and high-risk-score duplications indexed by pTS (β=-.047, p=1.26e-05); the association between overall psychopathology and pHI (β=0.031 p=4.20e-03); and the association between psychosis spectrum symptoms and pHI (β=0.038, p=1.19e-03) remained statistically significant after FDR correction for multiple comparisons. As also shown in eFigure 8, reported associations between PGS and neurodevelopmental outcomes, and between environmental factors and neurodevelopmental outcomes, were also robust to this sensitivity analysis. We note that the effect size was reduced for the association between CNV risk scores and cognitive outcomes when excluding known pathogenic CNVs from the sample – for deletions but not for duplications. This may be due to diminished statistical power to detect the true effect size when excluding 130 participants who disproportionately have high CNV risk scores – given previous evidence that the effect size of the association between CNV risk scores and general intelligence is similar whether or not known pathogenic CNVs were included in models [15,16]. For the neuroimaging analysis, 17 of the 59 participants in the main analysis with high CNV risk scores (pHI score or pTS score >1) had known pathogenic CNVs. Unsurprisingly, for a sensitivity analyses excluding participants with pathogenic CNVs, the association between CNV risk scores and brain deviation from the normative model dropped to "trend level" in terms of statistical significance (β=0.62, p=0.052) due to the reduced sampled size, though the effect size was undiminished (in fact marginally increased) compared to the observed association when known pathogenic CNVs were included in the model (original β=0.56, original p=0.039), supporting the conclusion that the association between CNV risk scores and brain deviation was similar whether or not CNVs were "pathogenic" in the sense of having known associations with cognition or psychopathology from prior literature. Future work investigating neuroimaging-CNV associations will benefit from larger samples or samples enriched for high CNV risk scores.

As described in the main results, we also performed a series of additional sensitivity analyses to assess the associations between CNV risk scores, cognition and psychopathology. First, an analysis including X chromosomal CNVs yielded similar outcomes compared to our main result (eTable 8). This sensitivity analysis could not include pHI because that score was derived for autosomal genes only, but the results for intolerance scores LOEUF and pLI were convergent with our main analyses. Second, an analysis including Affymetrix chips yielded an additional 442 participants with similar outcomes to the main results (eTable 9). Third, we modelled Illumina Chip Version as a random effect in statistical models of the association with outcome variables, showing highly convergent results with main analyses (e.g. pHI association with Overall Accuracy, β=-0.11, p=5.86e-25; pTS association with Overall Accuracy, β=-0.05, p=1.17-06; pHI association with psychosis spectrum symptoms, β=-0.49, p=3.49e-05) and suggesting that Chip Version did not confound the association between high-risk-score CNVs

© 2022 Alexander-Bloch A et al. *JAMA Psychiatry*.

and developmental outcomes (see eFigure 9). We also performed selected associations with outcome variables within Chip Version to further show that it was unlikely any biases between Chip Versions accounted for the reported results. Results were convergent with main analyses that combined data across Chip Version (e.g. eTable 12 shows results for the 5 most frequent Chip Versions, which together account for >90% of the data, for the association between CNV risk scores and total accuracy). Finally, main results were robust to sensitivity analyses that did not include covariates sex, self-identified race or ancestry PCs in models (eTable 11). All outcomes measures were age-normalized, but all results were also robust to including age as an additional regressor in statistical models.

© 2022 Alexander-Bloch A et al. *JAMA Psychiatry*.

eMethods 9. Analysis of family history

Mental illness in the parents of the present sample is likely to be related to outcome measures, as well as not only genetic but also environmental factors considered as predictor variables in the present study. For instance, parental history of psychiatric disorder is an indicator of genetic risk but is likely also to be associated with traumatic exposures. While information about parental history of mental illness *per se* is unfortunately not available in the PNC, a measure of family history of psychosis (in any first degree relative) was collected and described in prior work [58]. As would be predicted by prior work, this measure of family history was associated with traumatic exposures (β=0.11, p=5.96e-10) as well as neighborhood-level SES (β=0.03, p=0.00136). We reran models of environmental and genetic influence on outcomes including this measure of family history, and all results were robust to the inclusion of this measure in statistical models (eFigure 11). Moreover, there was an independent association between family history and overall psychopathology (β=0.06, p=6.39e-05). Speculatively, this supplemental analysis is consistent with the hypothesis that family history is related to but does not fully account for the environmental associations with neurodevelopmental outcomes reported in the present study. Limitations include the fact that this measure was focused on psychosis in particular and also that it was based on self- or caregiver-reported family history rather than independent clinical evaluation of family members. Further work, in studies designed to address these questions, is clearly required to investigate the combined influence of genetic and environmental effects on neurodevelopmental outcomes.

© 2022 Alexander-Bloch A et al. *JAMA Psychiatry*.

eFigure 1. Schematic of CNV data processing pipeline

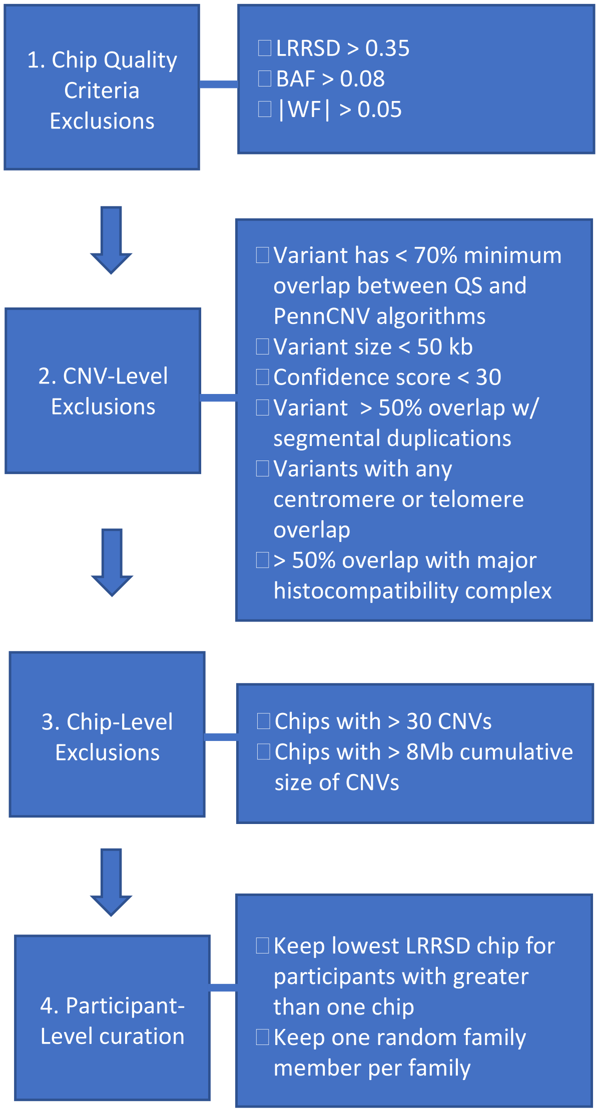

LRRSD, log r ratio standard deviation; BAF, B allele frequency; |WF|, absolute value of wave factor; QS, QuantiSNP

© 2022 Alexander-Bloch A et al. *JAMA Psychiatry*.

eFigure 2. Exclusion plot of chip frequency by number of CNV and markers

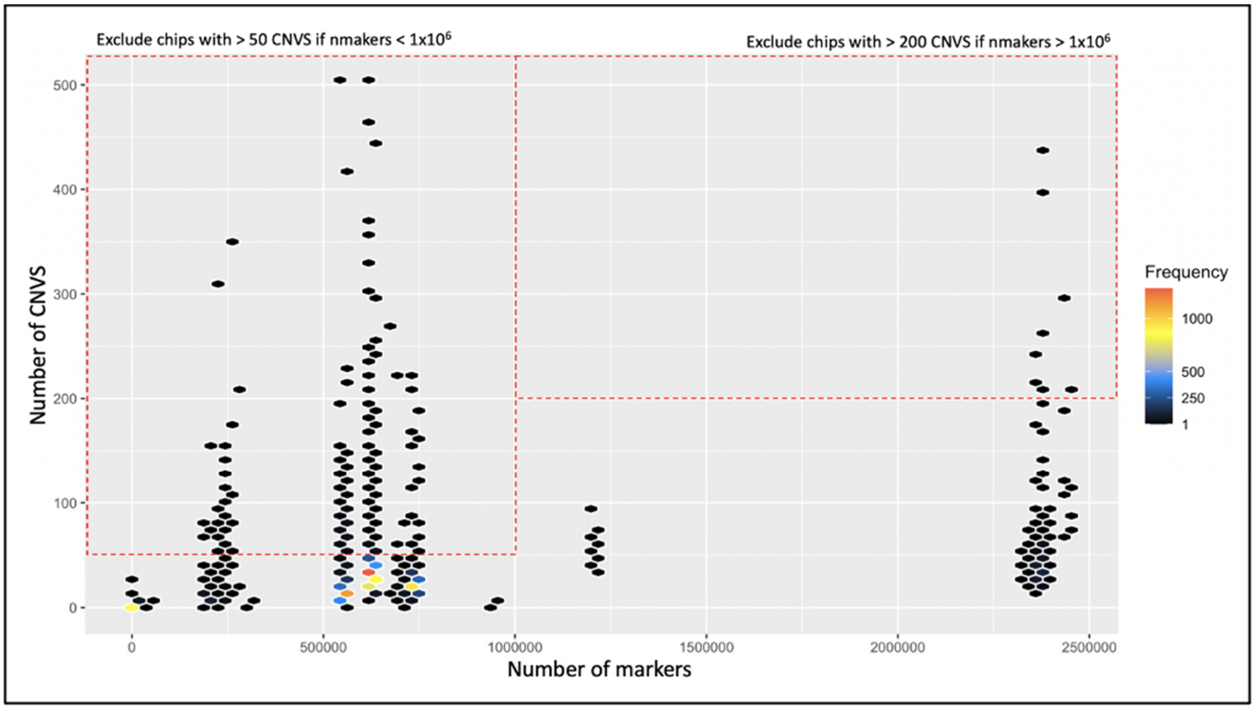

As part of chip quality control illustrated in eFigure 1, chips were assessed for their number of CNVs relative to their number markers (probes). Following 16, we excluded arrays with a suspiciously high number of CNVs detected (defined as ≥50 for low resolution arrays with <1 million probes, and ≥200 for high resolution arrays with ≥1 million probes). Red dotted boxes indicate catchment areas for exclusion, meaning that chips inside red boxes were excluded.

© 2022 Alexander-Bloch A et al. *JAMA Psychiatry*.

eFigure 3. Distribution of CNVs and CNV risk scores in the study sample

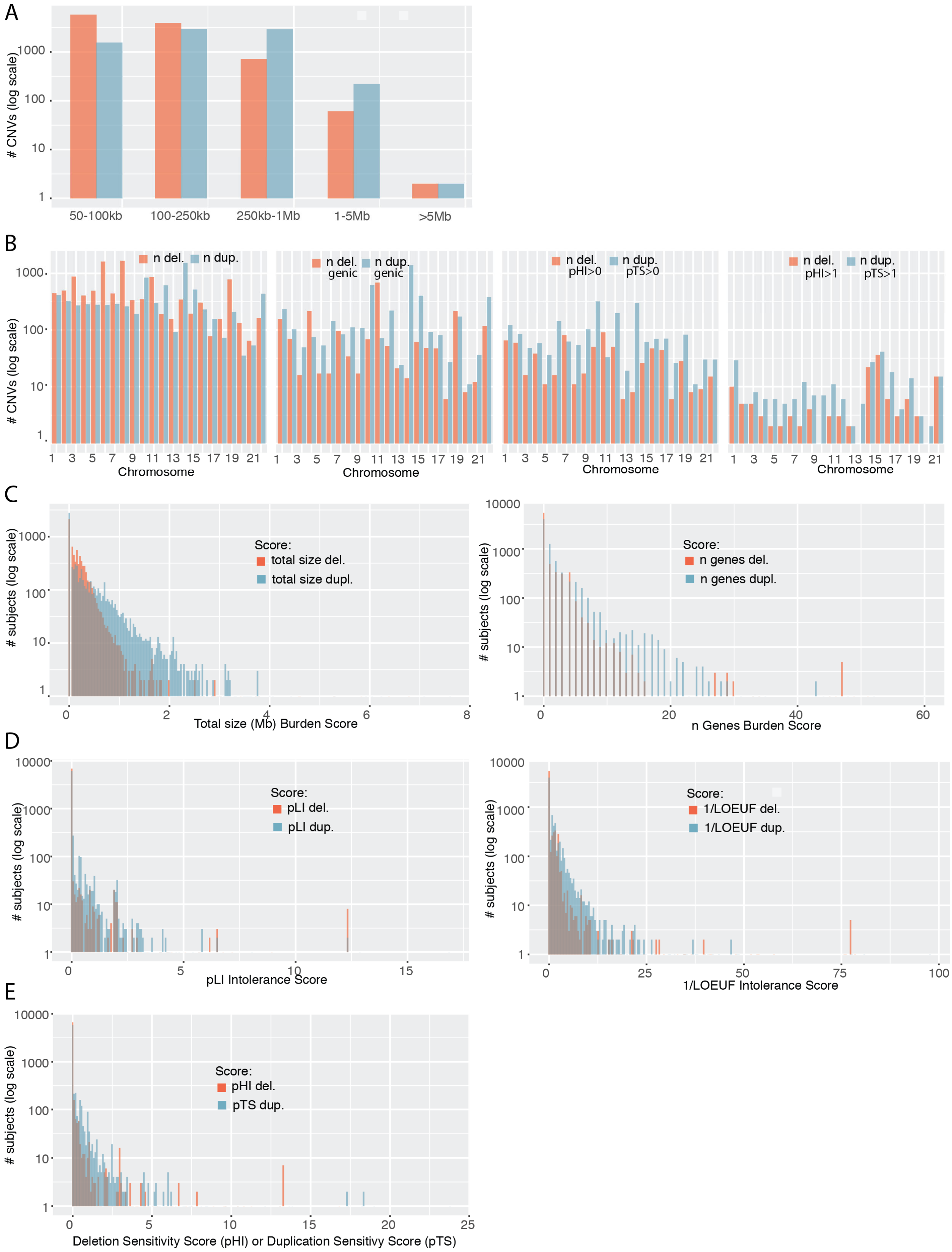

© 2022 Alexander-Bloch A et al. *JAMA Psychiatry*.

Distribution of CNVs and CNV risk scores in the study sample for deletions (red) and duplications (blue). All results are after the full quality control procedure described in eMethods 4, presented as histograms where the height of the lines corresponds to the number of CNVs (A-B) or to the number of participants (C-E). **A)** Total number of CNVs within 5 size bins. **B)** Total number of CNVs distributed across autosomes, from left to right: all CNVs; genic CNVs (encompassing at least one gene); CNVs with cumulative pHI or pTS score > 0; CNVs with cumulative pHI or pTS score > 1. Note this Panel contains the same information as Main Figure 1A presented in a different fashion. **C)** Participant burden scores: Left Panel, total size in Mb of all of a participant's CNVs; Right Panel, the number of genes fully encompassed by CNVs. **D)** Participant intolerance scores: Left Panel, the sum of genes' probability of loss intolerance (pLI) scores; Right Panel, the sum of the inverse of genes' loss of function observed/expected upper bound fraction (LOEUF) scores. **E)** Participant dosage sensitivity scores: the sum of genes' probability of haploinsuffiency (pHI) and probability of triplosensitivity (pTS) scores for deletions and duplications, respectively.

© 2022 Alexander-Bloch A et al. *JAMA Psychiatry*.

eFigure 4. CNV frequency generally decreases in association with increases in CNV risk score

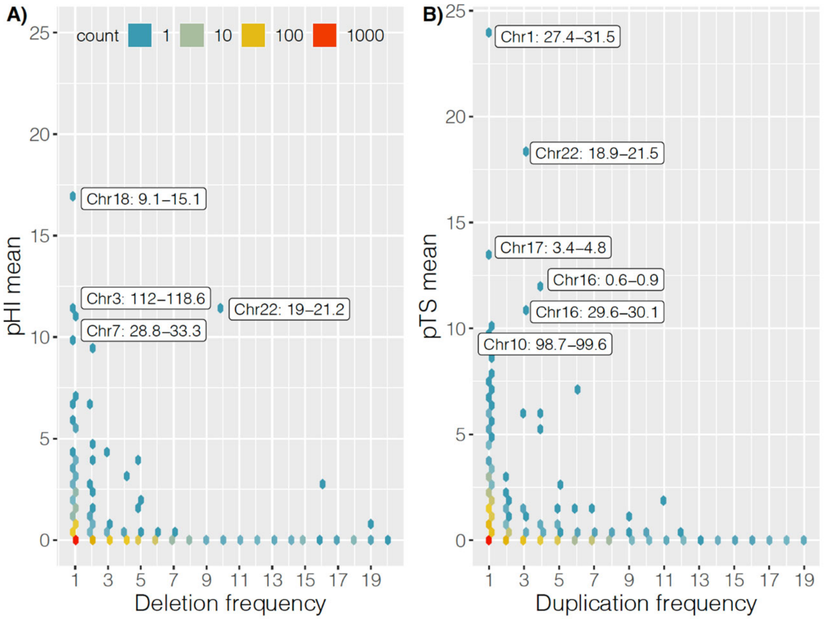

CNV frequency generally decreases in association with increases in CNV risk score (measured by cumulative probability of haploinsufficiency (pHI) in deletions and cumulative probability of triplosensitivity (pTS) in duplications. After applying quality control criteria outlined in eFigures 1-2 and eMethods 3, CNVs with 50% reciprocal overlap were considered members of the same CNV "cluster." A) CNV deletions shown with cumulative pHI of genes in each CNV cluster. B) CNV duplications shown with cumulative pTS of genes in each CNV cluster. In clusters, pHI or pTS was estimated as the mean of all CNVs in the cluster. Clusters with a mean pHI or pTS greater than 10 are labeled in the hexplot with their chromosome number and the mean start and stop (Mb) of the CNVs within the cluster. The x-axis shows the total number of participants with each CNV. As expected, most CNVs were unique (1-member clusters without 50% reciprocal overlap to another CNV) and had CNV risk scores close to 0. Note that clustering based on reciprocal overlap was performed for visualization purposes for this figure, but in all other reported analyses each participant's CNV risk score was calculated independently. Participant-level CNV risk scores were summed across all of a participant's deletions and duplications respectively. Note that this Figure shows CNV risk scores including known pathogenic CNVs, which were included in main results but excluded in sensitivity analysis (eMethods 8).

© 2022 Alexander-Bloch A et al. *JAMA Psychiatry*.

eFigure 5. CNV associations with cognition and psychopathology, including environmental factors in multiancestry sample

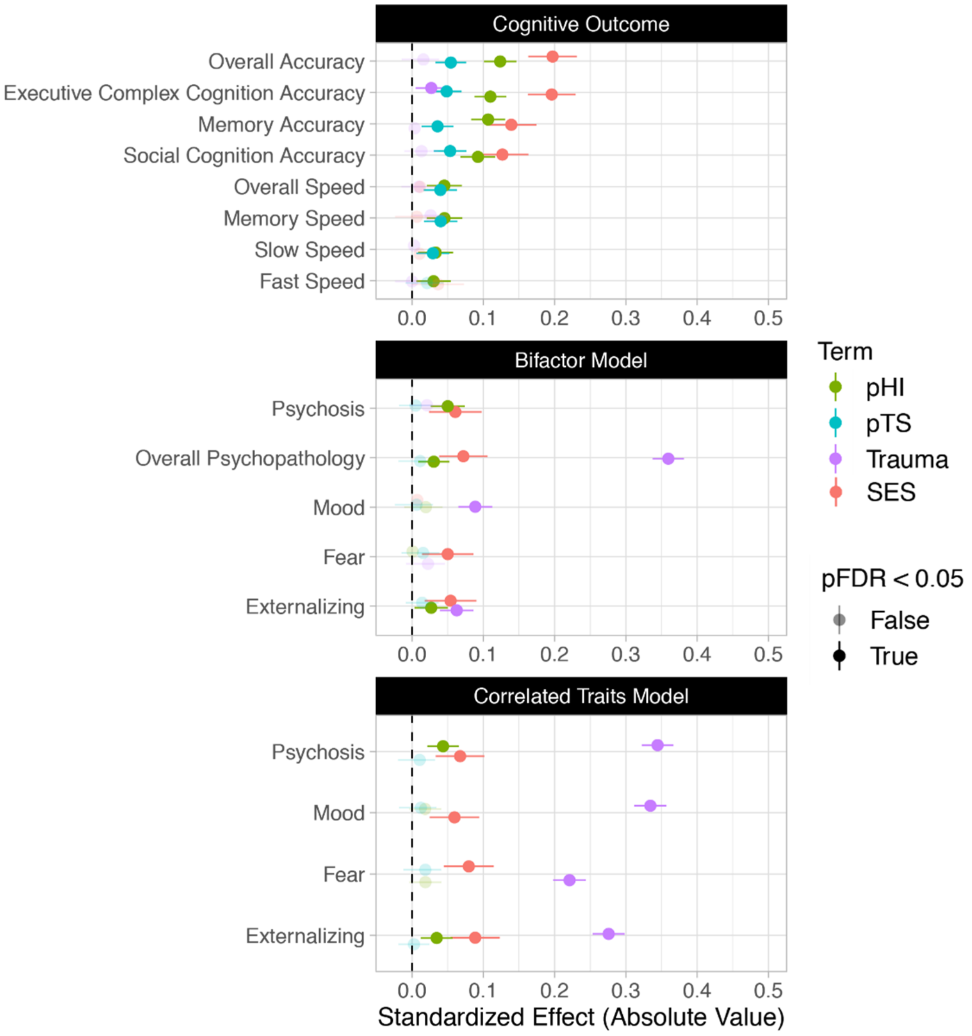

Analogous to Main Figure 1 but including environmental factors in addition to CNV risk scores. CNV risk scores were quantified as the cumulative probability of haploinsufficiency (pHI, a measure of sensitivity to deletion) or probability of triplosensitivity (pTS, a measure of sensitivity to duplication). Dot plots show the effect size (standardized beta coefficient) for the association of pHI and pTS with eight cognitive outcomes (Top Panel) including speed and accuracy scores for specific (e.g. "Memory", "Social Cognition") and global ("Overall") cognitive measures. "Slow speed" is summarized from items requiring deliberation, while "fast speed" is summarized from items requiring rapid decisions. For psychopathology outcomes, dot plots of the effect size (standardized beta coefficient) for pHI and pTS generated via bifactor models (Middle Panel) and correlated traits factor models (Bottom Panel). All outcome measures were age normalized, and additional covariates included self-identified race, sex and 10 ancestry principal components in all models. This figure was generated based on the multi-ancestry sample of participant genotyped on Illumina arrays that met quality control criteria (n=7,101), and p-values were corrected for 68 comparisons using the Benjamini-Hochberg false discovery rate (FDR).

© 2022 Alexander-Bloch A et al. *JAMA Psychiatry*.

eFigure 6. Combined models of developmental outcomes and their joint associations with CNV scores, environmental factors, and common variant polygenic scores (PGSs)

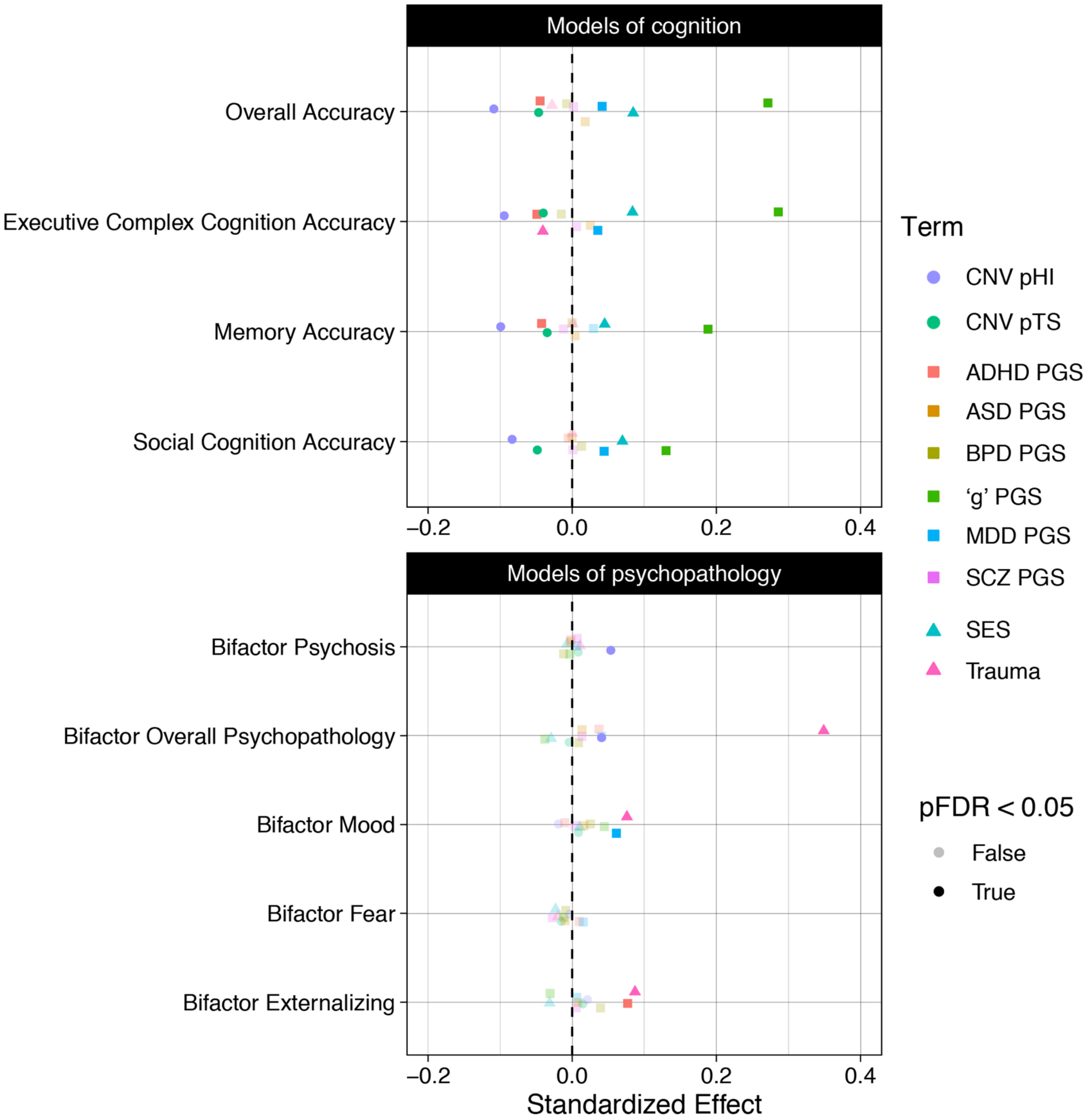

Analogous to Main Figure 2 but showing additional terms from the model and omitting depiction of 95% confidence intervals for clarity. Each point in the dot plots indicate the value of a given predictor variable's effect size in models of cognition or psychopathology for models of cognition (Top Panel) and models of psychopathology (Bottom Panel). CNV risk scores were quantified by deletion cumulative probability of haploinsufficiency (pHI) and duplication cumulative probability of triplosensitivity (pTS). Environmental factors comprised neighborhood socioeconomic status (SES) and trauma exposure. PGSs were included for ADHD, ASD, BPD, 'g' (general intelligence), MDD and SCZ. All outcome measures were age normalized, and additional covariates included self-identified race, sex and 10 ancestry principal components in all models. This analysis was conducted in the European ancestry sample genotyped with Illumina arrays that met quality control criteria (n=4,482) and p-values were corrected for 90 comparisons using the Benjamini-Hochberg false discovery rate (FDR).

© 2022 Alexander-Bloch A et al. *JAMA Psychiatry*.

eFigure 7. Brain imaging (MRI) deviation from normative models and CNV risk scores

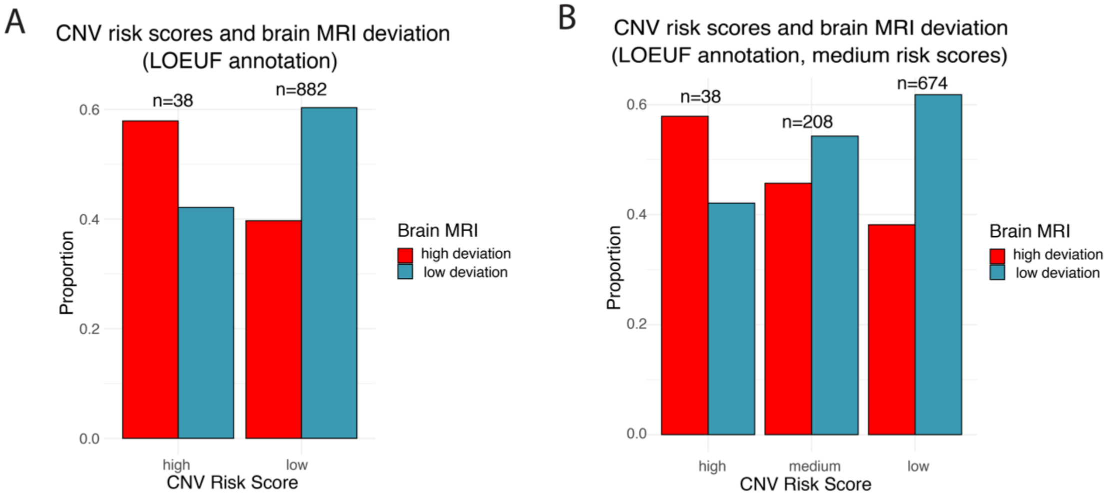

Analyses of the association between brain imaging (MRI) deviation from normative models and CNV risk scores using LEOUF-based risk score and incorporating a medium-risk-score category (compare with Main Figure 3). **A)** Proportion of individuals with high-risk-score CNVs (cumulative 1/LOEUF score > 2.85) who are categorized as low brain deviation (2nd to 9th decile in all imaging phenotypes) versus high brain deviation (1st or 10th decile in at least one imaging phenotype). **B)** Addition of medium-risk-score category (0 < cumulative 1/LOEUF ≤ 2.85). LOEUF, loss-of-function observed/expected upper bound fraction.

© 2022 Alexander-Bloch A et al. *JAMA Psychiatry*.

eFigure 8. Sensitivity analysis excluding participants with known pathogenic CNVs

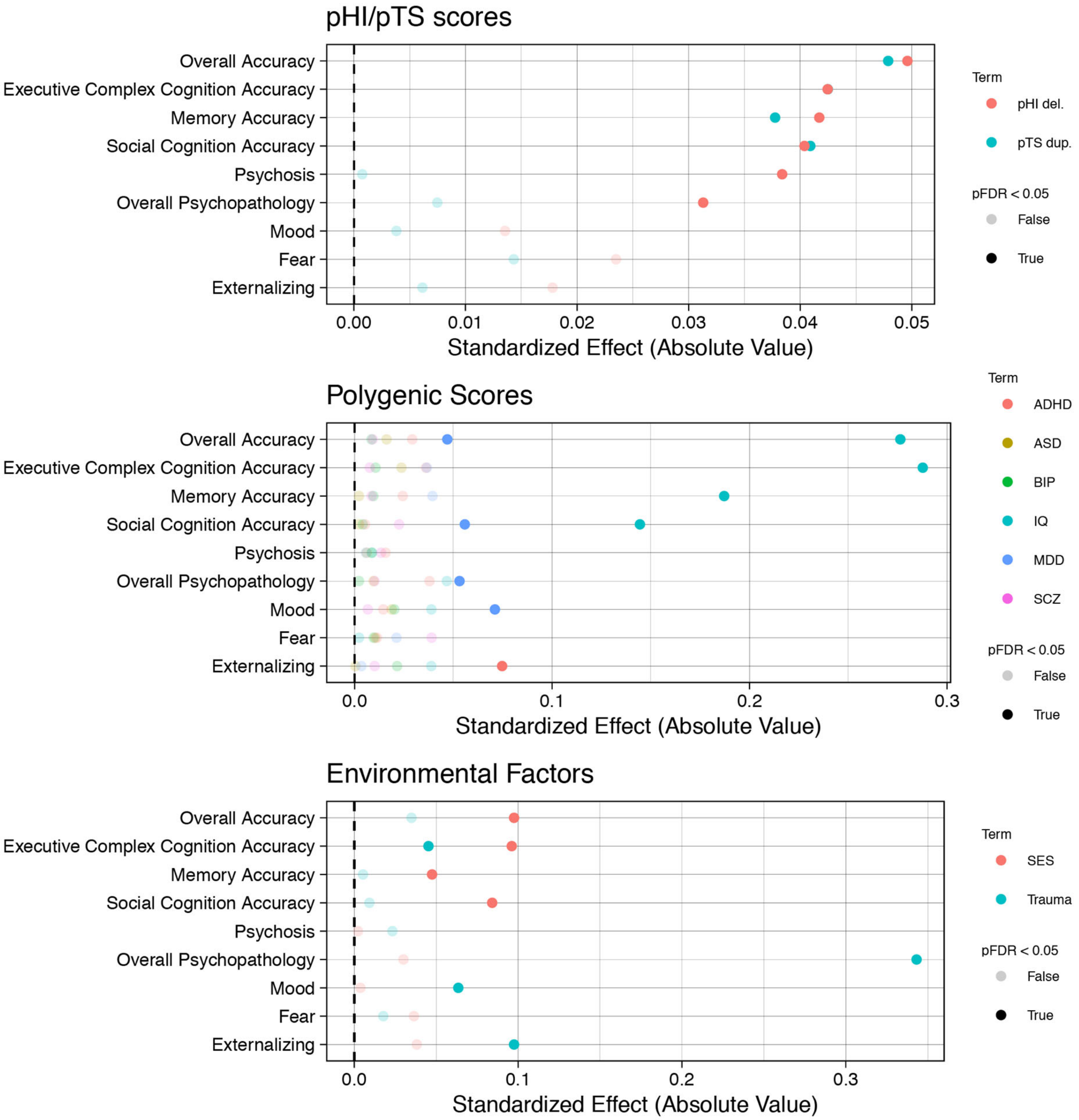

Results of sensitivity analysis excluding 130 total participants with 38 known pathogenic CNVs from the sample. The re-analysis of CNV risk score (Top Panel) was conducted in the multi-ancestry sample (n=7,101), while the analysis of polygenic scores (PGS, middle panel) was conducted in the European Ancestry sample (n=4,482). For consistency with Main Figure 2, the analysis of environmental factors (Bottom Panel) was also conducted in the European Ancestry sample (n=4,482). FDR-correction was across 90 total comparisons. All outcome measures were age normalized, and additional covariates included self-identified race, sex and 10 ancestry principal components in all models.

© 2022 Alexander-Bloch A et al. *JAMA Psychiatry*.

eFigure 9. Sensitivity analysis with random effect for Illumina Infinium Beadchip subversion

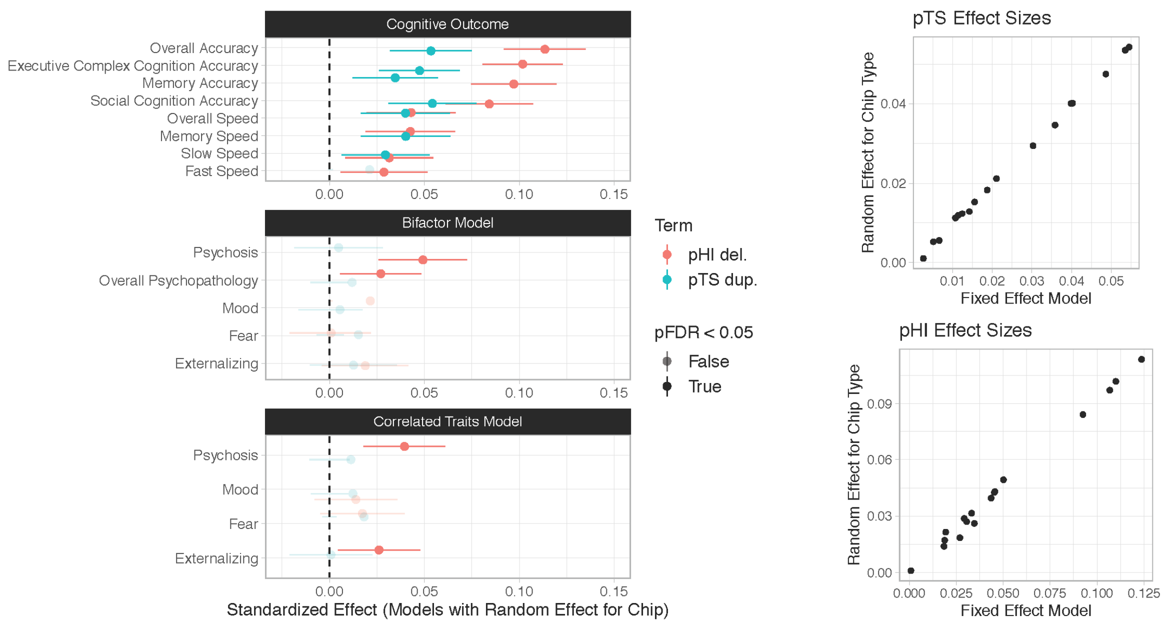

Results of sensitivity analysis incorporating a random effect for Illumina Infinium Beadchip subversion into statistical models (multi-ancestry sample size, n=7,101; FDR correction across 32 associations). Results were highly convergent with main analyses (compare with Main Figure 1). All outcome measures were age normalized, and additional covariates included self-identified race, sex and 10 ancestry principal components in all models.

© 2022 Alexander-Bloch A et al. *JAMA Psychiatry*.

eFigure 10. Full distributions of individual brain imaging–based centile scores

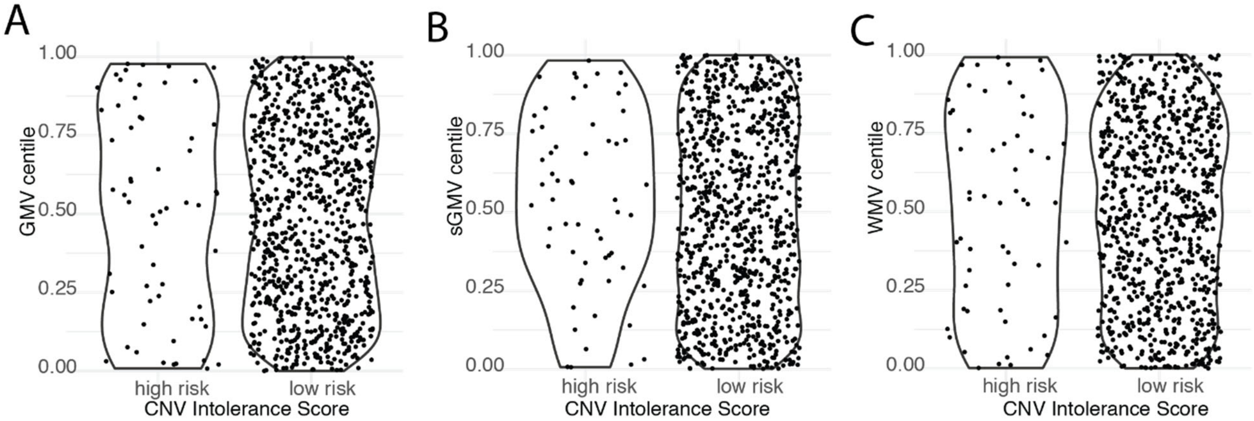

Illustration of full distributions of individual brain imaging based centile scores (age- and sex-stratified). Violin plots of individual brain imaging based centile scores: A) cortical gray matter volume (GMV), B) subcortical gray matter volume, C) cerebral white matter volume (WMV). CNV risk scores are determined as for Main Figure 3 (total pHI > 1 or total pTS > 1).

© 2022 Alexander-Bloch A et al. *JAMA Psychiatry*.

eFigure 11. Combined models of developmental outcomes and their joint associations with CNV scores, environmental factors, and common variant polygenic scores

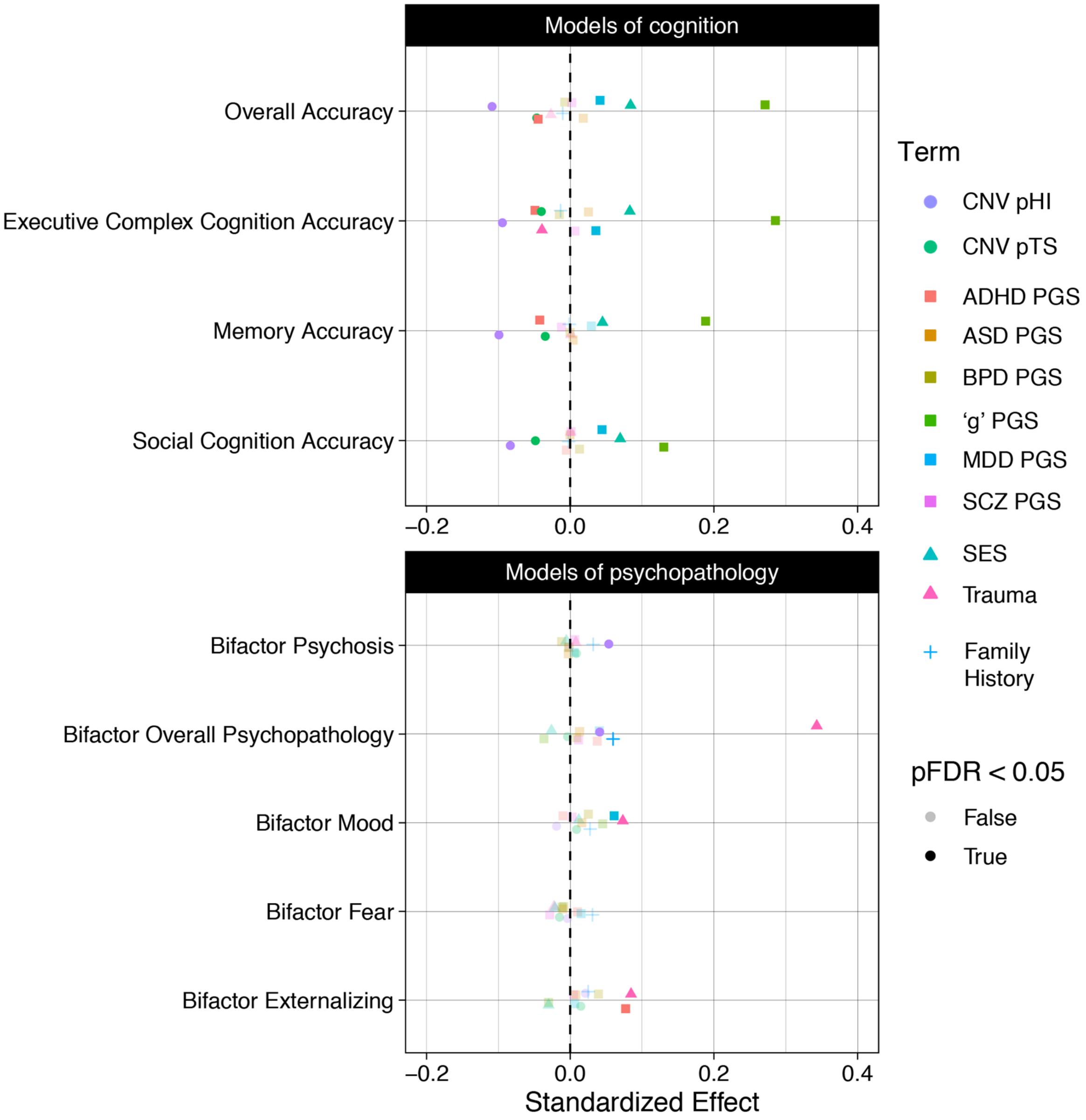

Analogous to eFigure 6 but in models that also include a measure of family of history of psychosis. Each point in the dot plots indicate the value of a given predictor variable's effect size in models of cognition or psychopathology for models of cognition (Top Panel) and models of psychopathology (Bottom Panel). CNV risk score is quantified by deletion cumulative probability of haploinsufficiency (pHI) and duplication cumulative probability of triplosensitivity (pTS). Environmental factors comprised neighborhood socioeconomic status (SES) and trauma exposure. PGSs were included for ADHD, ASD, BPD, 'g' (general intelligence), MDD and SCZ. All outcome measures were age normalized, and additional covariates included self-identified race, sex and 10 ancestry principal components in all models. This analysis was conducted in the European ancestry sample genotyped with Illumina arrays

© 2022 Alexander-Bloch A et al. *JAMA Psychiatry*.

that met quality control criteria (n=4,482) and p-values were corrected for 99 comparisons using the Benjamini-Hochberg false discovery rate (FDR).

© 2022 Alexander-Bloch A et al. *JAMA Psychiatry*.

eTable 1. Demographic information for CNV samples after quality control

**Demographic**	**All data**	**Illumina arrays**
Total n	7,543	7,101
Age	14.2 (3.66)	14.2 (3.67)
SES	0.06 (0.98)	0.125 (0.955)
Sex (Female)	3818 (50.6)	3574 (50.3%)
Ancestry		
European	4486 (59%)	4482 (63%)
African	2222 (30%)	1818 (26%)
Other	835 (11%)	801 (11%)

SES, Neighborhood socioeconomic status

© 2022 Alexander-Bloch A et al. *JAMA Psychiatry*.

eTable 2. Definitions of terms related to CNV risk scores

**CNV risk scores**	Quantitative measures of predicted risk based on annotating CNVs in terms of burden, intolerance or dosage sensitivity.
**Burden**	Measure of total size or number of genes encompassed by CNV.
**Intolerance**	Measure based on genes' intolerance to loss of function, for genes encompassed by a CNV (i.e. pLI and LOEUF scores).
**pLI**	Probability that a gene is intolerant to a loss of function mutation, where values closer to 1 are suggestive of intolerance [58].
**LOEUF**	Loss of function observed/expected upper bound fraction, a measure ranging from 0 to 2, where values below 0.35 are typically suggestive of intolerance [55].
**Dosage sensitivity**	Measure based on genes' deletion or duplication sensitivity, for genes encompassed by a CNV. The probability of haploinsufficiency (pHI) is measure of a gene's sensitivity to deletion, and the probability of triplosensitivity (pTS) is a measure of gene's sensitivity to duplication.[22]
**Pathogenic CNVs**	CNVs with specific, known clinical associations with cognition or psychopathology based on prior literature. CNVs with high risk scores are not necessarily pathogenic.

© 2022 Alexander-Bloch A et al. *JAMA Psychiatry*.

eTable 3. Logistic regression models of categorical diagnoses

Logistic regression models of categorical diagnoses (psychosis spectrum, ADHD, anxiety, and depression) and their association with CNV risk scores. Note that this table reports association between CNV risk scores and categorical diagnoses rather than dimensional psychopathology outcomes, which are the focus of main analyses. Covariates included sex, self-identified race, and 10 ancestry PCs. This table was generated from the multi-ancestry sample of participant genotyped on Illumina arrays that met quality control criteria (n=7101), and p-values were corrected for 8 comparisons using the Benjamini-Hochberg false discovery rate (FDR).

**Diagnosis**	**CNV Risk Score**	**Stand. β**	**95% CI**	**p-value**	**FDR-adj p**
Psychosis-spectrum	Deletion pHI	0.128	(0.056, 0.201)	0.000461	0.00369
	Duplication pTS	0.018	(-0.044, 0.074)	0.552	1
ADHD	Deletion pHI	0.110	(0.037, 0.182)	0.00260	0.0182
	Duplication pTS	0.020	(-0.050, 0.080)	0.546	1
Anxiety	Deletion pHI	0.042	(-0.027, 0.114)	0.242	1
	Duplication pTS	0.004	(-0.047, 0.057)	0.874	1
Depression	Deletion pHI	-0.062	(-0.178, 0.026)	0.230	1
	Duplication pTS	-0.049	(-0.200, 0.061)	0.453	1

pHI, cumulative probability of haploinsufficiency; pTS, cumulative triplosensitivity.

© 2022 Alexander-Bloch A et al. *JAMA Psychiatry*.

eTable 4. Models of cognitive and psychopathological outcomes

Models of cognitive and psychopathological outcomes and their association with CNV risk scores (cumulative pHI for deletions and pTS for duplications), environmental factors (neighborhood SES and traumatic exposures), and polygenic scores (PGSs for ASD, ADHD, SCZ, BPD, MDD, and intelligence). AIC and adjusted r^2 are shown for models with increasing complexity, from left to right: demographic covariates only (sex, self-identified race and 10 ancestry principal components); CNV risk scores and demographic covariates; environmental factors and demographic covariates; CNV risk scores, environmental factors, and demographic covariates; CNV risk scores, environmental factors, and demographic covariates. This table was generated from the European sample of participant genotyped on Illumina arrays that met quality control criteria (n=4,482). All outcome measures were age-normalized.

**Outcome**	**Demographic covariates**		**CNV risk scores**		**Environmental Factors**		**Environmental Factors and CNV risk scores**		**Environmental Factors, CNV risk scores, and PGSs**	
	**AIC**	***Adj r^2***	**AIC**	***Adj r^2***	**AIC**	***Adj r^2***	**AIC**	***Adj r^2***	**AIC**	***Adj r^2***
Overall Accuracy	11820	0.003	11750	0.019	11536	0.020	11461	0.037	11071	0.119
Executive Complex Cognition Accuracy	11693	0.007	11640	0.019	11438	0.026	11381	0.039	10933	0.133
Memory Accuracy	12067	0.006	12016	0.018	11829	0.010	11777	0.022	11602	0.062
Social Cognition Accuracy	12340	0.014	12299	0.024	12148	0.020	12107	0.029	12042	0.045
Overall Psychopathology	12304	0.003	12302	0.004	11647	0.141	11643	0.142	11620	0.147
Psychosis spectrum	12266	0.005	12256	0.007	11656	0.133	11643	0.136	11627	0.140
Externalizing	12226	0.010	12225	0.011	11778	0.106	11775	0.107	11714	0.120
Fear	12039	0.018	12040	0.018	11788	0.073	11789	0.073	11780	0.076
Mood	12465	0.006	12466	0.006	11942	0.117	11943	0.117	11919	0.123

CNV, copy number variant; AIC, Akaike information criterion; pHI, probability of haploinsufficiency; pTS, probability of triplosensitivity.

© 2022 Alexander-Bloch A et al. *JAMA Psychiatry*.

eTable 5. Exploratory analysis of interaction effects

Interaction effects were explored between CNV risk scores and environmental factors (neighborhood-level SES and trauma exposure) and between CNV risk scores and common variant risk indexed by polygenic scores. Statistics are shown for a subset of nominal effects (uncorrected $p<0.05$). No effects remain significant after FDR-correction for 144 multiple comparison. All models also included additive effects for pHI, pTS, PGS and environmental factors, self-reported race, sex and 10 ancestry principal components. This analysis was run on the European ancestry sample genotyped on Illumina chips that met quality control criteria (n=4,482)

Outcome	Interaction	Stand. beta	Lower 95% CI	Upper 95% CI	p-value	FDR-adj. p
Overall Accuracy	pHI:Trauma	-0.0370	-0.0706	-0.0035	0.0305	0.3656
Overall Accuracy	pTS:PGS.SCZ	-0.0367	-0.0668	-0.0066	0.0170	0.3095
Executive Complex Cognition Accuracy	pTS:SES	0.0225	0.0001	0.0449	0.0489	0.4400
Executive Complex Cognition Accuracy	pTS:PGS.SCZ	-0.0338	-0.0634	-0.0041	0.0257	0.3656
Social Cognition Accuracy	pHI:PGS.MDD	-0.0304	-0.0554	-0.0054	0.0172	0.3095
Social Cognition Accuracy	pTS:PGS.SCZ	-0.0461	-0.0798	-0.0125	0.0071	0.2645
Memory Accuracy	pHI:Trauma	-0.0423	-0.0779	-0.0067	0.0200	0.3204
Memory Accuracy	pTS:PGS.IQ	-0.0259	-0.0503	-0.0016	0.0365	0.3883
Mood Spectrum	pHI:SES	-0.0469	-0.0849	-0.0089	0.0156	0.3095
Mood Spectrum	pTS:PGS.IQ	-0.0392	-0.0666	-0.0118	0.0050	0.2645
Psychosis Spectrum	pHI:Trauma	0.0398	0.0023	0.0774	0.0378	0.3883
Psychosis Spectrum	pHI:PGS.BPD	0.0410	0.0110	0.0710	0.0073	0.2645
Psychosis Spectrum	pHI:PGS.SCZ	0.0498	0.0139	0.0856	0.0065	0.2645
Externalizing Spectrum	pTS:Trauma	0.0348	0.0015	0.0681	0.0406	0.3898
Externalizing Spectrum	pHI:PGS.MDD	0.0275	0.0029	0.0520	0.0283	0.3656
Externalizing Spectrum	pTS:PGS.ADHD	0.0401	0.0086	0.0717	0.0128	0.3095

SES, socioeconomic status; PGS, polygenic score; pHI, probability of haploinsuffiency; pTS, probability of triplosensitivity; CI, confidence interval

© 2022 Alexander-Bloch A et al. *JAMA Psychiatry*.

eTable 6. Known pathogenic CNVs identified in the PNC sample

Known pathogenic CNVs defined by systematic literature review identified in the sample. See eMethods 4; these CNVs were excluded in sensitivity analysis. Sorted by frequency (number of participants, n, shown in leftmost column). Cumulative probability of haploinsufficiency (pHI) and triplosensitivity (pTS) are defined as the sum of pHI and pTS of genes encompassed by CNVs for deletions or duplications, respectively. Start and stop are in hg19 coordinates.

n	CNV Region or Gene ID	Type	CHR	Start	Stop	Cumulative pHI	Cumulate pTS
20	15q11.2	DUP	chr15	22805313	23094530	-	1.86719
17	15q11.2	DEL	chr15	22805313	23094530	2.4473	-
10	22q11.2	DEL	chr22	19037332	21466726	12.87807	-
9	1q21.1 TAR	DUP	chr1	145394955	145807817	-	4.46906
8	NRXN1	DEL	chr2	50145643	51259674	0.97382	-
7	16p13.11	DUP	chr16	15511655	16293689	-	1.2352
6	ZMYM5	DUP	chr13	20411593	20437773	-	0.04618
5	16p12.1	DEL	chr16	21950135	22431889	2.07167	-
5	16p11.2 distal	DEL	chr16	28823196	29046783	4.57374	-
5	17p12 (HNPP/CMT1A)	DEL	chr17	14141387	15426961	1.65613	-
4	1q21.1 TAR	DEL	chr1	145394955	145807817	4.27452	-
4	1q21.1	DEL	chr1	146527987	147394444	2.04502	-
4	16p11.2 distal	DUP	chr16	28823196	29046783	-	5.68589
4	16p11.2 proximal	DUP	chr16	29650840	30200773	-	13.56827
4	17q12 HNF1B	DUP	chr17	34815904	36217432	-	6.04723
3	22q11.2	DUP	chr22	19037332	21466726	-	17.75638
2	15q11.2q13.1_BP2-BP3	DEL	chr15	22805313	28390339	7.66382	-
2	15q12 GABRB3 GABRA5	DEL	chr15	26971834	27548820	0.60443	-
2	16p11.2 proximal	DEL	chr16	29650840	30200773	7.37869	-
2	2q37	DEL	chr2	239716679	243199373	12.67625	-
2	DMRT1	DUP	chr9	841690	969090	-	0.04376
1	1q21.1	DUP	chr1	146527987	147394444	-	1.33485
1	10q11.21q11.23	DEL	chr10	49390199	51058796	3.76301	-
1	10q11.21q11.23	DUP	chr10	49390199	51058796	-	4.82419
1	15q11.2q13.1 BP2-BP3	DUP	chr15	22805313	28390339	-	6.11996
1	15q12 GABRB3 GABRA5	DUP	chr15	26971834	27548820	-	0.57187
1	15q13.1q13.3 BP3-BP5	DEL	chr15	29161368	32462776	3.93027	-
1	15q13.3 BP4-BP5 CHRNA7	DEL	chr15	31080645	32462776	2.63204	-
1	16p13.11	DEL	chr16	15511655	16293689	3.05968	-
1	16q23.3	DEL	chr16	82660399	83830215	0.50356	-
1	17q11.2 NF1	DEL	chr17	29107491	30265075	4.9606	-

© 2022 Alexander-Bloch A et al. *JAMA Psychiatry*.

1	2q13	DEL	chr2	111394040	112012649	1.01279	-
1	22q11.2 distal	DEL	chr22	21920127	23653646	4.20015	-
1	22q11.2 distal	DUP	chr22	21920127	23653646	-	7.03165
1	3q29	DEL	chr3	195720167	197354826	9.69381	-
1	4p16.3 (WH)	DUP	chr4	1552030	2091303	-	5.84965
1	VPS13B	DEL	chr8	100025494	100889808	0.74101	-

© 2022 Alexander-Bloch A et al. *JAMA Psychiatry*.

eTable 7. Association with CNV risk scores excluding known pathogenic CNVs

Association between CNV risk scores and overall cognitive accuracy, sorted by lowest AIC, showing the effect on models of excluding pathogenic CNVs. Overall accuracy scores were age-normalized, and additional covariates included sex, self-identified race and 10 ancestry principal components. This table was generated from the multi-ancestry sample of participant genotyped on Illumina arrays that met quality control criteria, and p-values were corrected for 16 comparisons using the Benjamini-Hochberg false discovery rate (FDR). This table is analogous to Main Table 1 but showing the effect of excluding pathogenic CNVs. To facilitate comparison, the original results including pathogenic CNVs are also appended below the double bar, with rows marked by asterisks (*), while the results for the sensitivity analysis excluding known pathogenic CNVs appear above the double bar.

CNV risk score	Deletion/ Duplication	Stand. β	95% CI	p-value	P (FDR)	Adj r^2	AIC
pHI	Deletion pHI<br>Duplication pTS	-0.050<br>-0.048	(-0.072, -0.027)<br>(-0.07, -0.026)	1.33E-05<br>1.80E-05	1.58E-03<br>2.12E-03	0.114	18578
pLI	Deletion pLI<br>Duplication pLI	-0.036<br>-0.055	(-0.059, -0.013)<br>(-0.077, -0.033)	2.30E-03<br>1.12E-06	2.41E-01<br>1.35E-04	0.113	18582
1/LOEUF	Deletion 1/LOEUF<br>Duplication 1/LOEUF	-0.039<br>-0.036	(-0.061, -0.016)<br>(-0.058, -0.014)	7.42E-04<br>1.29E-03	7.94E-02<br>1.37E-01	0.112	18593
Log pLI	Deletion log pLI<br>Duplication log pLI	-0.048<br>0.008	(-0.07, -0.026)<br>(-0.014, 0.031)	2.22E-05<br>4.59E-01	2.59E-03<br>1.00E+00	0.112	18597
N genes	Deletion n genes<br>Duplication n genes	-0.029<br>-0.039	(-0.051, -0.006)<br>(-0.061, -0.017)	1.24E-02<br>6.27E-04	1.00E+00<br>6.84E-02	0.112	18597
Total Size	Deletion Total Size<br>Duplication Total Size	-0.080<br>-0.024	(-0.16, -0.001)<br>(-0.076, 0.028)	4.76E-02<br>3.69E-01	1.00E+00<br>1.00E+00	0.110	18610
Log 1/LOEUF	Deletion log 1/LOEUF<br>Duplication log 1/LOEUF	-0.019<br>-0.014	(-0.042, 0.003)<br>(-0.036, 0.009)	8.82E-02<br>2.30E-01	1.00E+00<br>1.00E+00	0.110	18611
pHI>0 / pTS>0	Deletion pHI>0<br>Duplication pTS>0	-0.017<br>-0.006	(-0.039, 0.006)<br>(-0.029, 0.016)	1.39E-01<br>5.68E-01	1.00E+00<br>1.00E+00	0.110	18613
pHI*	Deletion pHI<br>Duplication pTS	-0.121<br>-0.054	(-0.144, -0.099)<br>(-0.076, -0.032)	7.41E-26<br>1.31E-06	9.49E-24<br>1.47E-04	0.125	18922
pLI*	Deletion pLI<br>Duplication pLI	-0.117<br>-0.059	(-0.14, -0.094)<br>(-0.081, -0.037)	1.03E-23<br>1.06E-07	1.30E-21<br>1.20E-05	0.124	18928
1/LOEUF*	Deletion 1/LOEUF<br>Duplication 1/LOEUF	-0.118<br>-0.044	(-0.14, -0.095)<br>(-0.066, -0.022)	2.94E-24<br>7.38E-05	3.73E-22<br>8.04E-03	0.123	18937
Log pLI*	Deletion log pLI<br>Duplication log pLI	-0.103<br>-0.046	(-0.126, -0.081)<br>(-0.068, -0.025)	2.90E-19<br>3.28E-05	3.63E-17<br>3.61E-03	0.121	18959
N gene*	Deletion n genes<br>Duplication n genes	-0.092<br>-0.022	(-0.114, -0.069)<br>(-0.044, 0.000)	1.85E-15<br>4.86E-02	2.29E-13<br>1.00E+00	0.117	18989
Total Size*	Deletion Total Size<br>Duplication Total Size	-0.084<br>0.001	(-0.106, -0.062)<br>(-0.021, 0.023)	1.28E-13<br>9.14E-01	1.57E-11<br>1.00E+00	0.115	19001
Log 1/LOEUF*	Deletion log 1/LOEUF<br>Duplication log 1/LOEUF	-0.066<br>-0.013	(-0.089, -0.044)<br>(-0.035, 0.009)	7.07E-09<br>2.51E-01	8.06E-07<br>1.00E+00	0.113	19021
pHI>0 / * pTS>0	Deletion pHI>0<br>Duplication pTS>0	-0.162<br>-0.032	(-0.238, -0.085)<br>(-0.084, 0.019)	3.24E-05<br>2.20E-01	3.60E-03<br>1.00E+00	0.111	19038

CNV, copy number variant; AIC, Akaike information criterion; CI, confidence interval; pLI, probability of loss intolerance; 1/LOEUF, inverse of loss-of-function observed/expected upper bound fraction; pHI, probability of haploinsufficiency; pTS, probability of triplosensitivity; *rows below the double bar refer to main results including known pathogenic CNVs, whereas rows above the double bar refer to results from the sensitivity analysis that excluded known pathogenic CNVs.

© 2022 Alexander-Bloch A et al. *JAMA Psychiatry*.

eTable 8. Associations with CNV risk scores including X chromosome CNVs

Associations between CNV risk scores and overall cognitive accuracy including X chromosome CNVs. Rows are sorted by lowest AIC. Overall accuracy scores were age-normalized, and additional covariates included sex, self-identified race and 10 ancestry principal components. Analogous to Main Table 1 but including CNVs on the X chromosome. This table was generated from the multi-ancestry sample of participant genotyped on Illumina arrays that met quality control criteria, and p-values were corrected for 16 comparisons using the Benjamini-Hochberg false discovery rate (FDR).

	CNV score	Stand. β	95% CI	p-value	P (FDR)	Adj r^2	AIC
pHI	Deletion pHI<br>Duplication pTS	-0.122<br>-0.06	(-0.145, -0.099)<br>(-0.082, -0.037)	2.54E-25<br>1.46E-07	3.25E-23<br>1.69E-05	0.123	18351
pLI	Deletion pLI<br>Duplication pLI	-0.098<br>-0.042	(-0.121, -0.075)<br>(-0.064, -0.02)	2.04E-16<br>2.37E-04	2.59E-14<br>2.61E-02	0.116	18406
1/LOEUF	Deletion 1/LOEUF<br>Duplication 1/LOEUF	-0.078<br>-0.042	(-0.102, -0.055)<br>(-0.064, -0.019)	5.47E-11<br>2.61E-04	6.89E-09<br>2.85E-02	0.113	18431
Log pLI	Deletion log pLI<br>Duplication log pLI	-0.075<br>-0.032	(-0.098, -0.052)<br>(-0.055, -0.01)	2.42E-10<br>5.20E-03	2.95E-08<br>5.20E-01	0.112	18438
N genes	Deletion n genes<br>Duplication n genes	-0.055<br>-0.045	(-0.078, -0.031)<br>(-0.068, -0.022)	4.07E-06<br>9.84E-05	4.64E-04<br>1.09E-02	0.11	18450
Total Size	Deletion Total Size<br>Duplication Total Size	-0.18<br>-0.057	(-0.259, -0.1)<br>(-0.112, -0.002)	9.54E-06<br>4.21E-02	1.08E-03<br>1.00E+00	0.108	18463
Log 1/LOEUF	Deletion log 1/LOEUF<br>Duplication log 1/LOEUF	-0.048<br>-0.026	(-0.071, -0.025)<br>(-0.049, -0.003)	5.46E-05<br>2.48E-02	6.12E-03<br>1.00E+00	0.108	18464
pHI>0 / pTS>0	Deletion pHI>0<br>Duplication pTS>0	-0.054<br>-0.008	(-0.077, -0.031)<br>(-0.031, 0.015)	3.67E-06<br>4.81E-01	4.22E-04<br>1.00E+00	0.108	18465

CNV, copy number variant; AIC, Akaike information criterion; CI, confidence interval; pLI, probability of loss intolerance; 1/LOEUF, inverse of loss-of-function observed/expected upper bound fraction; pHI, probability of haploinsufficiency; pTS, probability of triplosensitivity

© 2022 Alexander-Bloch A et al. *JAMA Psychiatry*.

eTable 9. Associations with CNV risk scores including Affymetrix arrays

Association between CNV risk scores and overall cognitive accuracy including Affymetrix arrays. With Affymetrix array, total n=7,543. Rows sorted by lowest AIC. Overall accuracy scores were age-normalized, and additional covariates included sex, self-identified race and 10 ancestry principal components. Analogous to Main Table 1 but including Affymetrix arrays for the multi-ancestry sample of participant that met quality control criteria. p-values were corrected for 16 comparisons using the Benjamini-Hochberg false discovery rate (FDR).

	**CNV score**	**Stand. β**	**95% CI**	**p-value**	**P (FDR)**	**Adj r^2**	**AIC**
pHI	Deletion pHI<br>Duplication pTS	-0.121<br>-0.054	(-0.142, -0.099)<br>(-0.075, -0.033)	6.33E-27<br>6.28E-07	8.10E-25<br>7.04E-05	0.13	20016
pLI	Deletion pLI<br>Duplication pLI	-0.116<br>-0.061	(-0.138, -0.093)<br>(-0.082, -0.039)	2.34E-24<br>2.27E-08	2.95E-22<br>2.56E-06	0.129	20023
1/LOEUF	Deletion 1/LOEUF<br>Duplication 1/LOEUF	-0.116<br>-0.046	(-0.138, -0.094)<br>(-0.067, -0.025)	6.89E-25<br>1.93E-05	8.76E-23<br>2.11E-03	0.128	20032
Log pLI	Deletion log pLI<br>Duplication log pLI	-0.103<br>-0.05	(-0.125, -0.081)<br>(-0.071, -0.028)	2.72E-20<br>4.81E-06	3.41E-18<br>5.29E-04	0.126	20051
N genes	Deletion n genes<br>Duplication n genes	-0.09<br>-0.024	(-0.112, -0.068)<br>(-0.046, -0.003)	9.65E-16<br>2.57E-02	1.20E-13<br>1.00E+00	0.122	20087
Total Size	Deletion Total Size<br>Duplication Total Size	-0.082<br>-0.002	(-0.103, -0.06)<br>(-0.023, 0.019)	1.21E-13<br>8.59E-01	1.49E-11<br>1.00E+00	0.12	20102
Log 1/LOEUF	Deletion log 1/LOEUF<br>Duplication log 1/LOEUF	-0.068<br>-0.017	(-0.09, -0.046)<br>(-0.038, 0.005)	1.15E-09<br>1.29E-01	1.31E-07<br>1.00E+00	0.118	20117
pHI>0 / pTS>0	Deletion pHI>0<br>Duplication pTS>0	-0.179<br>-0.036	(-0.254, -0.104)<br>(-0.087, 0.014)	3.11E-06<br>1.60E-01	3.45E-04<br>1.00E+00	0.116	20133

CNV, copy number variant; AIC, Akaike information criterion; CI, confidence interval; pLI, probability of loss intolerance; 1/LOEUF, inverse of loss-of-function observed/expected upper bound fraction; pHI, probability of haploinsufficiency; pTS, probability of triplosensitivity

eTable 10. No association between pHI CNV risk scores and demographic variables

**Biological Sex**	pHI mean	*t*-statistic	p-value
Male | Female	0.09 | 0.08	0.85	0.40
**Self-identified race**	pHI mean	*t*-statistic	p-value
European American | African American	0.08 | 0.08	0.16	0.88
European American | Other	0.08 | 0.10	0.66	0.51
African American | Other	0.08 | 0.10	0.72	0.47
**Age**	Pearson's r	t-statistic	p-value
	-0.01	0.72	0.47

pHI, probability of haploinsufficiency

© 2022 Alexander-Bloch A et al. *JAMA Psychiatry*.

eTable 11. Associations without demographic covariates included in statistical models

Association between CNV risk scores and overall cognitive accuracy without demographic covariates included in statistical models. Overall accuracy scores were age-normalized. Analogous to Main Table 1 but without demographic covariates included in statistical models. This table was generated from the multi-ancestry sample of participant genotyped on Illumina arrays that met quality control criteria, and p-values were corrected for 16 comparisons using the Benjamini-Hochberg false discovery rate (FDR).

	CNV score	Stand. β	95% CI	p-value	P (FDR)	Adj r^2	AIC
pHI	Deletion pHI	-0.120	(-0.143, -0.096)	1.19E-22	2.85E-21	0.017	19726
	Duplication pTS	-0.060	(-0.083, -0.037)	3.76E-07	5.32E-06		
pLI	Deletion pLI	-0.116	(-0.141, -0.092)	4.16E-21	9.14E-20	0.017	19730
	Duplication pLI	-0.065	(-0.088, -0.042)	3.16E-08	5.05E-07		
1/LOEUF	Deletion 1/LOEUF	-0.117	(-0.141, -0.093)	1.61E-21	3.70E-20	0.016	19737
	Duplication 1/LOEUF	-0.054	(-0.077, -0.03)	5.57E-06	6.69E-05		
Log pLI	Deletion log pLI	-0.106	(-0.13, -0.083)	2.16E-18	4.55E-17	0.014	19748
	Duplication log pLI	-0.058	(-0.081, -0.035)	1.00E-06	1.31E-05		
N genes	Deletion n genes	-0.096	(-0.119, -0.072)	2.42E-15	4.83E-14	0.011	19770
	Duplication n genes	-0.047	(-0.07, -0.024)	8.05E-05	8.86E-04		
Total Size	Deletion Total Size	-0.089	(-0.112, -0.065)	1.26E-13	2.40E-12	0.009	19785
	Duplication Total Size	-0.028	(-0.051, -0.004)	1.94E-02	3.88E-02		
Log 1/LOEUF	Deletion log 1/LOEUF	-0.071	(-0.095, -0.048)	2.53E-09	4.56E-08	0.006	19802
	Duplication log 1/LOEUF	-0.036	(-0.059, -0.012)	2.60E-03	1.86E-02		
pHI>0 / pTS>0	Deletion pHI>0	-0.237	(-0.318, -0.157)	7.20E-09	1.22E-07	0.005	19811
	Duplication pTS>0	-0.054	(-0.109, 0.001)	5.29E-02	5.29E-02		

CNV, copy number variant; AIC, Akaike information criterion; CI, confidence interval; pLI, probability of loss intolerance; 1/LOEUF, inverse of loss-of-function observed/expected upper bound fraction; pHI, probability of haploinsufficiency; pTS, probability of triplosensitivity

© 2022 Alexander-Bloch A et al. *JAMA Psychiatry*.

eTable 12. Impact of Illumina beadchip subversion

Assessing the impact of Illumina Chip Version on the association between CNV risk score and developmental outcomes. Table shows results for overall accuracy within chip for the 5 most frequent chips which together account for >90% of the sample. Overall accuracy scores were age-normalized, and additional covariates included sex, self-identified race and 10 ancestry principal components. Analogous to Main Table 1 but showing models of data from each chip separately. The row "all chip" also shows data from the full sample for comparison. This table was generated from the multi-ancestry sample of participant genotyped on Illumina arrays that met quality control criteria. P-values shown in this table were not corrected for multiple comparisons.

	**Chip sample size**	**Stand. β**	**95% CI**	**p-value**
pHI				
	2,784	-0.0954	(-0.139,-0.0518)	1.86E-05
	1,329	-0.195	(-0.247,-0.143)	3.61E-13
	1,274	-0.163	(-0.214,-0.112)	6.57E-10
	752	-0.068	(-0.139,0.00333)	0.0617
	266	-0.162	(-0.281,-0.0433)	0.00771
	**All Chips**	**-0.122**	**(-0.145,-0.0992)**	**7.41E-26**
pTS				
	2,784	-0.0514	(-0.0863,-0.0518)	0.00389
	1,329	-0.0665	(-0.118,-0.143)	0.0119
	1,274	-0.0682	(-0.12,-0.112)	0.00949
	752	-0.0131	(-0.0843,0.00333)	0.718
	266	-0.0313	(-0.15,-0.0433)	0.603
	**All Chips**	**0.054**	**(-0.0759,-0.0322)**	**1.31E-06**

© 2022 Alexander-Bloch A et al. *JAMA Psychiatry*.

eReferences.

1. Calkins ME, Moore TM, Merikangas KR, et al. The psychosis spectrum in a young US community sample: findings from the Philadelphia Neurodevelopmental Cohort. *World Psychiatry*. 2014;13(3):296-305.
2. Satterthwaite TD, Elliott MA, Ruparel K, et al. Neuroimaging of the Philadelphia neurodevelopmental cohort. *Neuroimage*. 2014;86:544-553.
3. Moore TM, Reise SP, Gur RE, Hakonarson H, Gur RC. Psychometric properties of the Penn Computerized Neurocognitive Battery. *Neuropsychology*. 2015;29(2):235-246.
4. Gur RC, Richard J, Hughett P, et al. A cognitive neuroscience-based computerized battery for efficient measurement of individual differences: standardization and initial construct validation. *J Neurosci Methods*. 2010;187(2):254-262.
5. Kaufman J, Birmaher B, Brent D, et al. Schedule for Affective Disorders and Schizophrenia for School-Age Children-Present and Lifetime Version (K-SADS-PL): Initial Reliability and Validity Data. *Journal of the American Academy of Child & Adolescent Psychiatry*. 1997;36(7):980-988. doi:10.1097/00004583-199707000-00021
6. Moore TM, Calkins ME, Satterthwaite TD, et al. Development of a computerized adaptive screening tool for overall psychopathology (“p”). *J Psychiatr Res*. 2019;116:26-33.
7. Caspi A, Houts RM, Belsky DW, et al. The p factor: One general psychopathology factor in the structure of psychiatric disorders? *Clin Psychol Sci*. Published online 2014. doi:10.1177/2167702613497473
8. Lahey BB, Applegate B, Hakes JK, Zald DH, Hariri AR, Rathouz PJ. Is there a general factor of prevalent psychopathology during adulthood? *J Abnorm Psychol*. 2012;121(4):971-977.
9. Allegrini AG, Cheesman R, Rimfeld K, et al. The p factor: genetic analyses support a general dimension of psychopathology in childhood and adolescence. *J Child Psychol Psychiatry*. 2020;61(1):30-39.
10. Calkins ME, Moore TM, Satterthwaite TD, et al. Persistence of psychosis spectrum symptoms in the Philadelphia Neurodevelopmental Cohort: a prospective two-year follow-up. *World Psychiatry*. 2017;16(1):62-76.
11. Alexander-Bloch AF, Raznahan A, Shinohara RT, et al. The architecture of co-morbidity networks of physical and mental health conditions in military veterans. *medRxiv*. Published online 2020.
12. Benjamini Y, Hochberg Y. Controlling the False Discovery Rate: A Practical and Powerful Approach to Multiple Testing. *J R Stat Soc Series B Stat Methodol*. 1995;57(1):289-300.
13. Grice JW. Computing and evaluating factor scores. *Psychol Methods*. 2001;6(4):430-450.
14. Moore TM, Martin IK, Gur OM, et al. Characterizing social environment’s association with neurocognition using census and crime data linked to the Philadelphia Neurodevelopmental Cohort. *Psychological Medicine*. 2016;46(3):599-610. doi:10.1017/s0033291715002111
15. Huguet G, Schramm C, Douard E, et al. Measuring and estimating the effect sizes of copy number variants on general intelligence in community-based samples. *JAMA Psychiatry*. Published online 2018. doi:10.1001/jamapsychiatry.2018.0039
16. Huguet G, Schramm C, Douard E, et al. Genome-wide analysis of gene dosage in 24,092 individuals estimates that 10,000 genes modulate cognitive ability. *Mol Psychiatry*. Published online January 7, 2021. doi:10.1038/s41380-020-00985-z
17. Colella S, Yau C, Taylor JM, et al. QuantiSNP: an Objective Bayes Hidden-Markov Model to detect and accurately map copy number variation using SNP genotyping data. *Nucleic Acids Res*. 2007;35(6):2013-2025.
18. Wang K, Li M, Hadley D, et al. PennCNV: an integrated hidden Markov model designed for high-resolution copy number variation detection in whole-genome SNP genotyping data. *Genome Res*. 2007;17(11):1665-1674.

© 2022 Alexander-Bloch A et al. *JAMA Psychiatry*.

19. Xu L, Hou Y, Bickhart DM, Song J, Liu GE. Comparative analysis of CNV calling algorithms: Literature survey and a case study using bovine high-density SNP data. *Microarrays (Basel)*. 2013;2(3):171-185.
20. Marioni JC, Thorne NP, Valsesia A, et al. Breaking the waves: improved detection of copy number variation from microarray-based comparative genomic hybridization. *Genome Biol*. 2007;8(10):R228.
21. Sanders SJ, He X, Willsey AJ, et al. Insights into Autism Spectrum Disorder Genomic Architecture and Biology from 71 Risk Loci. *Neuron*. 2015;87(6):1215-1233.
22. Collins RL, Glessner JT, Porcu E, et al. A cross-disorder dosage sensitivity map of the human genome. *medRxiv*. Published online 2021. https://www.medrxiv.org/content/10.1101/2021.01.26.21250098v1.full
23. Coe BP, Witherspoon K, Rosenfeld JA, et al. Refining analyses of copy number variation identifies specific genes associated with developmental delay. *Nat Genet*. 2014;46(10):1063-1071.
24. Cooper GM, Coe BP, Girirajan S, et al. A copy number variation morbidity map of developmental delay. *Nat Genet*. 2011;43(9):838-846.
25. Moreno-De-Luca D, Sanders SJ, Willsey AJ, et al. Using large clinical data sets to infer pathogenicity for rare copy number variants in autism cohorts. *Mol Psychiatry*. 2013;18(10):1090-1095.
26. Stefansson H, Meyer-Lindenberg A, Steinberg S, et al. CNVs conferring risk of autism or schizophrenia affect cognition in controls. *Nature*. 2014;505(7483):361-366.
27. Marshall CR, Howrigan DP, Merico D, et al. Contribution of copy number variants to schizophrenia from a genome-wide study of 41,321 subjects. *Nat Genet*. 2017;49(1):27-35.
28. Fox J. *Applied Regression Analysis and Generalized Linear Models*. 3rd ed. SAGE Publications; 2015.
29. Hilbe JM. Poisson Regression. In: *Modeling Count Data*. Cambridge University Press; 2014:35-73.
30. Schultz LM, Merikangas AK, Ruparel K, et al. Stability of Polygenic Scores Across Discovery Genome-Wide Association Studies. *bioRxiv*. Published online June 18, 2021:2021.06.18.449060. doi:10.1101/2021.06.18.449060
31. Chang CC, Chow CC, Tellier LC, Vattikuti S, Purcell SM, Lee JJ. Second-generation PLINK: rising to the challenge of larger and richer datasets. *Gigascience*. 2015;4:7.
32. Manichaikul A, Mychaleckyj JC, Rich SS, Daly K, Sale M, Chen WM. Robust relationship inference in genome-wide association studies. *Bioinformatics*. 2010;26(22):2867-2873.
33. Misc Functions of the Department of Statistics, Probability Theory Group (Formerly: E1071), TU Wien [R package e1071 version 1.7-6]. Published online March 18, 2021. Accessed April 17, 2021. https://CRAN.R-project.org/package=e1071
34. Ge T, Chen CY, Ni Y, Feng YCA, Smoller JW. Polygenic prediction via Bayesian regression and continuous shrinkage priors. *Nat Commun*. 2019;10(1):1776.
35. Vilhjálmsson BJ, Yang J, Finucane HK, et al. Modeling linkage disequilibrium increases accuracy of polygenic risk scores. *Am J Hum Genet*. 2015;97(4):576-592.
36. Lloyd-Jones LR, Zeng J, Sidorenko J, et al. Improved polygenic prediction by Bayesian multiple regression on summary statistics. *Nat Commun*. 2019;10(1):5086.
37. Ni G, Zeng J, Revez JA, et al. A comparison of ten polygenic score methods for psychiatric disorders applied across multiple cohorts. *Biol Psychiatry*. 2021;90(9):611-620.
38. Demontis D, Walters RK, Martin J, et al. Discovery of the first genome-wide significant risk loci for attention deficit/hyperactivity disorder. *Nat Genet*. 2019;51(1):63-75.
39. Grove J, Ripke S, Als TD, et al. Identification of common genetic risk variants for autism spectrum disorder. *Nat Genet*. 2019;51(3):431-444.
40. Howard DM, Adams MJ, Clarke TK, et al. Genome-wide meta-analysis of depression identifies 102 independent variants and highlights the importance of the prefrontal brain regions. *Nat Neurosci*. 2019;22(3):343-352.

© 2022 Alexander-Bloch A et al. *JAMA Psychiatry*.

41. Stahl EA, Breen G, Forstner AJ, et al. Genome-wide association study identifies 30 loci associated with bipolar disorder. *Nat Genet*. 2019;51(5):793-803.
42. Sullivan P. scz2021. Published online May 26, 2021. doi:10.6084/M9.FIGSHARE.14672178.V1
43. Schizophrenia Working Group of the Psychiatric Genomics Consortium, Ripke S, Walters JTR, O'Donovan MC. Mapping genomic loci prioritises genes and implicates synaptic biology in schizophrenia. *Genetic and Genomic Medicine*. Published online September 13, 2020. doi:10.1101/2020.09.12.20192922
44. Savage JE, Jansen PR, Stringer S, et al. Genome-wide association meta-analysis in 269,867 individuals identifies new genetic and functional links to intelligence. *Nat Genet*. 2018;50(7):912-919.
45. Rosen AFG, Roalf DR, Ruparel K, et al. Quantitative assessment of structural image quality. *Neuroimage*. 2018;169:407-418.
46. Moreau CA, Ching CR, Kumar K, Jacquemont S, Bearden CE. Structural and functional brain alterations revealed by neuroimaging in CNV carriers. *Curr Opin Genet Dev*. 2021;68:88-98.
47. Pietschnig J, Penke L, Wicherts JM, Zeiler M, Voracek M. Meta-analysis of associations between human brain volume and intelligence differences: How strong are they and what do they mean? *Neurosci Biobehav Rev*. 2015;57:411-432.
48. Haijma SV, Van Haren N, Cahn W, Koolschijn PCMP, Hulshoff Pol HE, Kahn RS. Brain Volumes in Schizophrenia: A Meta-Analysis in Over 18 000 Subjects. *Schizophr Bull*. Published online October 2012. http://eutils.ncbi.nlm.nih.gov/entrez/eutils/elink.fcgi?dbfrom=pubmed&id=23042112&retmode=ref&cmd=prlinks
49. Butler MG, Dasouki MJ, Zhou XP, et al. Subset of individuals with autism spectrum disorders and extreme macrocephaly associated with germline PTEN tumour suppressor gene mutations. *J Med Genet*. 2005;42(4):318-321.
50. Shinawi M, Liu P, Kang SHL, et al. Recurrent reciprocal 16p11.2 rearrangements associated with global developmental delay, behavioural problems, dysmorphism, epilepsy, and abnormal head size. *J Med Genet*. 2010;47(5):332-341.
51. Brunetti-Pierri N, Berg JS, Scaglia F, et al. Recurrent reciprocal 1q21.1 deletions and duplications associated with microcephaly or macrocephaly and developmental and behavioral abnormalities. *Nat Genet*. 2008;40(12):1466-1471.
52. Modenato C, Martin-Brevet S, Moreau CA, et al. Lessons learnt from neuroimaging studies of Copy Number Variants, a systematic review. *Biol Psychiatry*. Published online 2021. https://www.biologicalpsychiatryjournal.com/article/S0006-3223(21)01394-9/abstract
53. Bethlehem RAI, Seidlitz J, White SR, et al. Brain charts for the human lifespan. *bioRxiv*. Published online June 10, 2021:2021.06.08.447489. doi:10.1101/2021.06.08.447489
54. Lv J, Di Biase M, Cash RFH, et al. Individual deviations from normative models of brain structure in a large cross-sectional schizophrenia cohort. *Mol Psychiatry*. Published online September 22, 2020. doi:10.1038/s41380-020-00882-5
55. Karczewski KJ, Francioli LC, Tiao G, et al. The mutational constraint spectrum quantified from variation in 141,456 humans. *Nature*. 2020;581(7809):434-443.
56. Kraemer HC, Blasey CM. *How Many Subjects?* 2nd ed. SAGE Publications; 2015.
57. Taylor JH, Asabere N, Calkins ME, et al. Characteristics of youth with reported family history of psychosis spectrum symptoms in the Philadelphia Neurodevelopmental Cohort. *Schizophr Res*. 2020;216:104-110.
58. Lek M, Karczewski KJ, Minikel EV, et al. Analysis of protein-coding genetic variation in 60,706 humans. *Nature*. 2016;536(7616):285-291.

© 2022 Alexander-Bloch A et al. *JAMA Psychiatry*.
